# A Scoping Review on Simulation-Based Design Optimization in Marine Engineering: Trends, Best Practices, and Gaps

**DOI:** 10.1007/s11831-024-10127-1

**Published:** 2024-05-15

**Authors:** Andrea Serani, Thomas P. Scholcz, Valentina Vanzi

**Affiliations:** 1 National Research Council-Institute of Marine Engineering, Rome, Italy; 2 Maritime Research Institute Netherlands, Wageningen, The Netherlands; 3 Department of Biomedicine and Prevention, University of Rome Tor Vergata, Rome, Italy

## Abstract

This scoping review assesses the current use of simulation-based design optimization (SBDO) in marine engineering, focusing on identifying research trends, methodologies, and application areas. Analyzing 277 studies from Scopus and Web of Science, the review finds that SBDO is predominantly applied to optimizing marine vessel hulls, including both surface and underwater types, and extends to key components like bows, sterns, propellers, and fins. It also covers marine structures and renewable energy systems. A notable trend is the preference for deterministic single-objective optimization methods, indicating potential growth areas in multi-objective and stochastic approaches. The review points out the necessity of integrating more comprehensive multidisciplinary optimization methods to address the complex challenges in marine environments. Despite the extensive application of SBDO in marine engineering, there remains a need for enhancing the methodologies’ efficiency and robustness. This review offers a critical overview of SBDO’s role in marine engineering and highlights opportunities for future research to advance the field.

## Introduction

Simulation-based design optimization (SBDO), also known as simulation-driven design optimization (SDDO), has emerged as a critical tool in marine engineering, profoundly impacting various aspects of the field. This approach, which integrates numerical solutions with computer-aided design (CAD) software and optimization algorithms, empowers engineers to refine performance, cost-efficiency, and safety in marine structures, including ships, underwater vehicles, offshore platforms, and notably, marine energy production systems.

Traditional marine engineering practices, reliant on empirical data and heuristic approaches, often face limitations in adaptability and precision. These methods, though time-tested, struggle to cope with the increasing complexity of marine engineering challenges, especially in the face of stringent environmental regulations and the demand for higher efficiency. SBDO addresses these challenges by enabling a more nuanced exploration of design possibilities, leveraging computational power to identify optimal solutions that balance performance, cost, and environmental considerations.

In ship hull design, SBDO replaces traditional methods, which are heavily reliant on experience and trial-and-error approaches. By analyzing hydrodynamic performance across different hull designs, SBDO enables the optimization of shape and dimensions, thus reducing drag and enhancing fuel efficiency [1–3].

For marine propulsion systems, SBDO is invaluable in dealing with the complexity of various components like engines, propellers, shafts, and rudders. It facilitates the optimization of these components for maximum efficiency and reduced fuel consumption [4–12].

A pivotal area where SBDO is making significant strides is in the development and optimization of marine energy production systems. As the world increasingly seeks sustainable energy sources, marine energy systems, such as tidal [13–21] and wave energy converters [22–25], have gained prominence. SBDO plays a crucial role in designing these systems to maximize energy extraction and efficiency while ensuring resilience to marine environmental challenges. The optimization of these systems is vital for advancing renewable energy technologies and contributes significantly to sustainable marine practices.

Additionally, SBDO enhances the safety and reliability of marine structures. For offshore structures [26], which face harsh environmental conditions, SBDO is instrumental in evaluating and improving structural integrity under various scenarios.

Looking ahead, the field of SBDO in marine engineering is poised for significant advancements. Emerging trends like the integration of machine learning algorithms and the incorporation of real-time data analytics are expected to further revolutionize SBDO applications. These advancements will not only refine the optimization process but also open new avenues for addressing complex, multifaceted marine engineering challenges. This scoping review aims to present a comprehensive, current overview of SBDO in marine engineering, highlighting its applications and pointing to future research directions within marine and ocean engineering contexts.

**Fig. 1:**
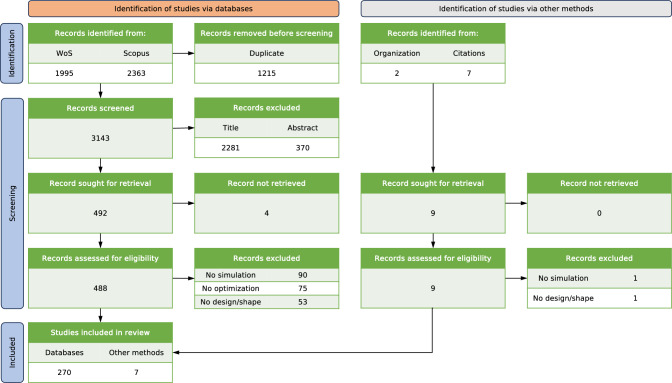
PRISMA flow chart

## Scoping Review Methodology

Due to a noticeable increase in research output and the proliferation of primary research over the past few years, the need to systematically identify and synthesize the existing literature has become mandatory in research. This critical issue has first arisen in clinical medicine but nowadays it represents a priority in many other disciplines including engineering [27]. Scoping reviews are extremely useful to accomplish this goal. The original framework for conducting scoping reviews was proposed by Arksey and O’Malley [28] and further extended by Joanna Briggs Institute (JBI) Collaboration in 2015 [29]. Recently, the JBI Scoping Reviews Methodology Group formally defined scoping reviews as a “*type of evidence synthesis that aims to systematically identify and map the breadth of evidence available on a particular topic, field, concept, or issue, often irrespective of source (i.e., primary research, reviews, non-empirical evidence) within or across particular contexts*” [30]. Despite other review methods, scoping reviews use a broader approach for mapping literature and addressing a broader research question without performing articles’ quality assessment [31].

### Research Questions

Central to this review is the exploration of current best practices in SBDO applied to marine engineering. This inquiry is structured into three fundamental questions:

1. What are the primary aims and approaches in the existing literature on SBDO methods in marine engineering, and how do they compare?
2. What issues are encountered when applying SBDO methods to marine engineering problems?
3. What are the main research gaps and potential future directions in this field?

### Inclusion and Exclusion Criteria

The inclusion criteria for the articles in this review were meticulously defined to ensure a focused and relevant collection of literature. Articles were selected based on their direct relevance to SBDO applications in marine engineering. This included studies demonstrating the use of SBDO in practical marine engineering projects, theoretical advancements in SBDO methods specific to marine applications, and reviews of SBDO methods within the marine engineering context.

Exclusion criteria were equally stringent to maintain the review’s scope and quality. Articles not directly related to SBDO, such as those focusing on general design optimization without a clear simulation-based component, were excluded. Studies outside the realm of marine engineering, or those employing SBDO in a manner not applicable to marine engineering challenges, were also omitted. Furthermore, non-peer-reviewed articles, such as conference abstracts/papers and editorials, were excluded to ensure the review’s academic rigor.

### Databases and Keywords

Web of Science (WoS) and Scopus were chosen as the primary databases for their extensive coverage of interdisciplinary scientific literature, ensuring a comprehensive collection of relevant articles in marine engineering and optimization. These databases are renowned for their rigorous indexing of high-quality, peer-reviewed academic journals, which aligns with the review’s emphasis on academic rigor.

The bibliographic search strategy was carefully designed to capture the broad scope of SBDO research in marine engineering, employing a combination of keywords specifically targeted within the titles, abstracts, and keywords sections (TITLE-ABS-KEY) of articles. The chosen keywords aimed to include a comprehensive range of studies relevant to the field: (“Simulation*” OR “Computation*”) AND (“Optimi*”) AND (“Design*” OR “Shape*” OR “Form*”) AND (“Ship*” OR “Hull” OR “Vessel” OR “Marine” OR “Ocean”). This strategic choice ensured the inclusion of pertinent research while maintaining a focused scope on SBDO applications within marine engineering.

**Fig. 2:**
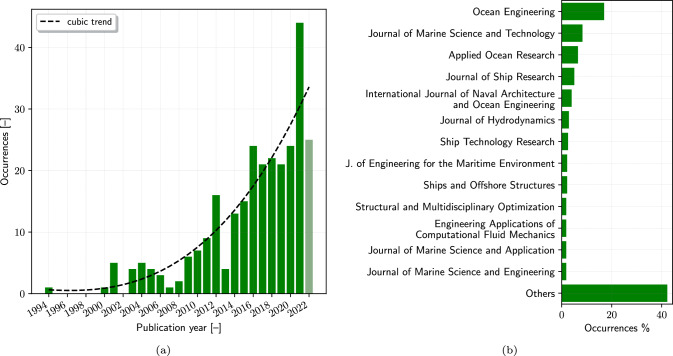
Publications trend (**a**) and journals occurrences (**b**)

### Search Procedure

The preferred reporting items for systematic reviews statement extended to scoping reviews (PRISMA-ScR) are used as reporting guidelines [32]. The PRISMA flow diagram (see Fig. 1) meticulously outlines the process undertaken for the selection of articles in the present scoping review. The articles search was conducted on August 1st, 2022, with no restriction on the date of publication and type of study, but considering only journal papers written in English. The diagram begins with the identification phase, where 3143 records were sourced through WoS and Scopus, indicating a comprehensive initial search strategy. Reference lists of all included articles were scanned to look for literature that had not been obtained previously.

Subsequent stages in the diagram reflect the screening and eligibility assessment processes. Notably, a significant number of records were excluded during the initial screening, likely due to title (2281) and abstract (370) relevance checks. This highlights the precision of our inclusion criteria, ensuring that only the most pertinent articles were considered (492) for full-text review.

The eligibility phase, as depicted, involved a more detailed review of the full texts, leading to further exclusion of articles that did not meet the specific criteria set for this review. These criteria were crucial in filtering out articles that did not include simulation, optimization strategies, or design/shape optimization.

Finally, the included studies (277), as shown in the diagram, represent a curated collection of articles that passed through this rigorous selection process, ensuring a high degree of relevance and quality in the research articles selected for this review.

**Fig. 3:**
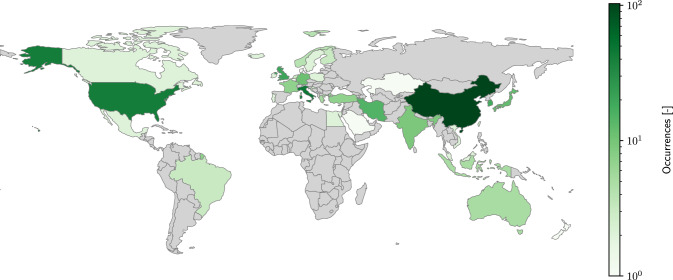
Publications occurrences geographical distribution (absolute value per country on a logarithmic scale)

## Results

The following subsections delineate the comprehensive findings of the scoping review, focusing on the key developments and trends within the realm of SBDO in marine engineering. This analysis aims to distill a broad spectrum of research efforts into discernible patterns, offering insights into the evolution, current practices, and future directions in the field. By examining a variety of aspects, from publication trends and journal distributions to the nuanced details of optimization techniques and application areas, this section endeavors to provide a holistic understanding of the state-of-the-art in SBDO as applied to marine engineering.

It may be noted that different terms have been used interchangeably to describe the overarching process of integrating computational simulations with design optimization in marine engineering. While SBDO and SDDO are prevalent, the analysis reveals both their widespread use and nuanced differences. SBDO emerges as the most comprehensive term, encompassing the full spectrum of leveraging simulation tools for optimizing design parameters. This terminology aligns with the holistic approach of using simulations to inform and drive the optimization process, where the objective is to enhance design performance metrics while navigating through the constraints imposed by complex marine engineering challenges. On the other hand, SDDO often highlights the initial stages of the design process, where simulations guide the conceptual and preliminary design decisions before formal optimization techniques are applied. This term underscores the importance of simulations in shaping the design space and influencing early design choices, which are crucial for setting the stage for subsequent optimization. The review suggests that while these terms broadly address the same domain of integrating simulations with optimization, they can reflect different focuses or stages within the broader SBDO process. This distinction is vital for understanding the scope and emphasis of various studies within the field, as well as for appreciating the multifaceted nature of SBDO in marine engineering.

Figure 2a illustrates a chronological trend in the number of publications per year on the topic. Starting from 1994, the year of the first publication retrieved on the topic [1], a noticeable increase in publications can be observed over the years (specifically starting from 2009), indicating a growing interest and advancement in the field. It’s important to note that the data for the year 2022 is partial, as the bibliographic research was conducted on August 1, 2022. This uptick reflects the evolving complexity and significance of SBDO in addressing contemporary challenges in marine engineering. The progressive increase underscores the technology’s rising relevance, potentially correlating with advancements in computational capabilities and the growing demand for efficient, optimized marine systems.

**Fig. 4:**
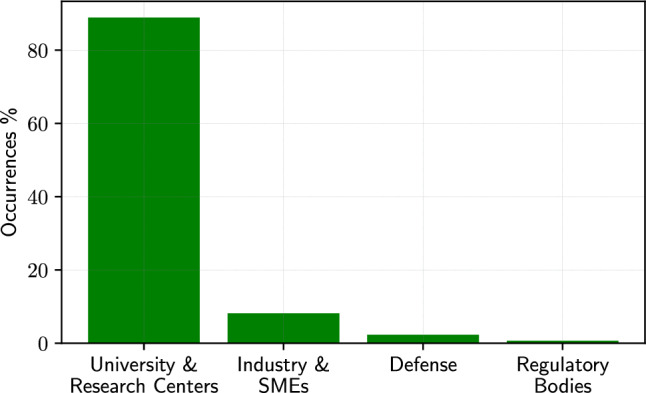
Publications origin occurrences by entity

Figure 2b presents a distribution of publications across various journals, highlighting those with the highest frequency of articles. Overall the Ocean Engineering journal covers 17.2% of the overall publications, whereas the other journals all contain less than 10% of the publications on SBDO. Moreover, the category ’Others’ encapsulates a range of journals that individually contribute to less than 2% of total publications, signifying a wide dissemination of research in this field across diverse scientific platforms. This distribution not only reflects the interdisciplinary nature of the field but also points to the key academic outlets that are central to the dissemination of SBDO research.

Based on a detailed analysis of the distribution and contributions, the results offer intriguing insights into global research trends and collaborative dynamics. The geographical distribution (see Fig. 3) showcases a significant concentration of contributions from China, accounting for 29.3% of the papers reviewed, with a diverse representation from 48 different entities. This is followed by Italy (13.9%), the United States (11.9%), the United Kingdom (5.7%), South Korea (5.1%), Iran (4.3%), Japan (3.4%), and Germany (3.1%), highlighting a global interest and varied focus across these regions. The predominance of university and research centers contributions, with 89% of the instances (see Fig. 4), signifies the academic inclination of SBDO research, whereas the industry and small and medium enterprises (SMEs), defense agencies, and regulatory bodies’ engagement, though lesser in number, underscore the multi-sectoral relevance of SBDO applications in marine engineering. This diverse geographical and institutional representation underscores the universal appeal and applicability of SBDO techniques across different marine engineering challenges, reflecting a rich picture of research efforts aimed at advancing marine technology and sustainability. The data suggest a vibrant and collaborative research ecosystem, with significant contributions emerging from both academia and industry, pointing towards an integrated approach to innovation in marine engineering through SBDO.

The following subsections present a categorization of SBDO research into several key areas, resulting in a systematic description of the vast body of work in this domain. The examination begins with problem formulation strategies, identifying the complex nature and challenges of the design optimizations present in the various studies. Subsequent analysis delves into the parameterization techniques used in SBDO. The focus then shifts to the solvers utilized in SBDO and optimization strategies. Finally, a deeper discussion of the applications is given.

**Fig. 5:**
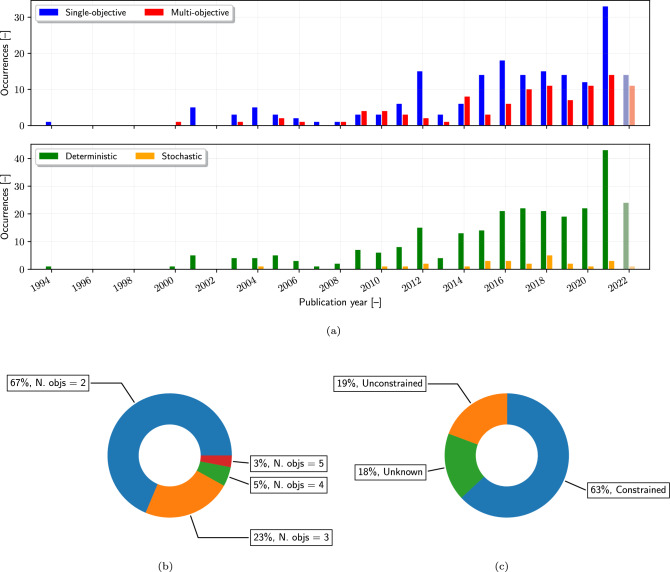
Problem formulation: (**a**) occurrences by year (top) single- versus multi-objective and (bottom) deterministic versus stochastic; (**b**) number of objectives overall occurrences for multi-objective problems; (**c**) use of constraints overall occurrences

**Fig. 6:**
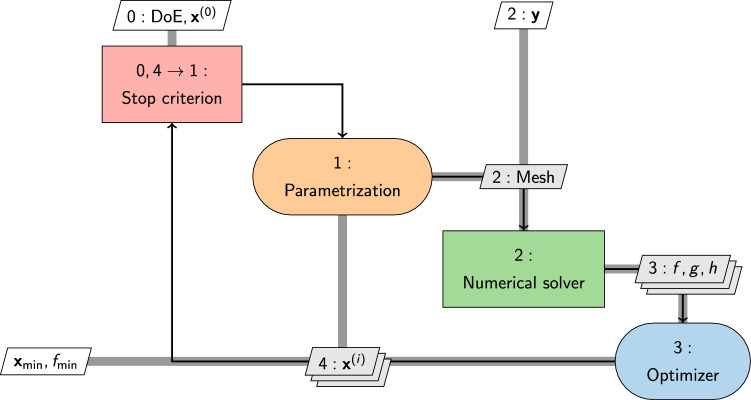
Overview of a general SBDO process through the XDSM diagram

### Problem Formulations

The field of SBDO in marine engineering exhibits a range of problem formulations, from straightforward deterministic single-objective optimization to more complex multi-objective and stochastic optimization approaches. The evolution towards embracing these complexities is gradual, reflecting a preference for simpler, more intuitive methods (see Fig. 5).

Central to the SBDO approach is the deterministic single-objective optimization, which remains predominant due to its clear and straightforward formulation:

1 $$\begin{aligned} \min _{{\textbf{x}}} \quad&f({\textbf{x}},{\textbf{y}})\nonumber \\ \text {subject to} \quad&g_i({\textbf{x}},{\textbf{y}}) \le 0, \quad i = 1, \ldots , m \nonumber \\ \text {and to} \quad&h_j({\textbf{x}},{\textbf{y}}) = 0, \quad j = 1, \ldots , p \nonumber \\ \text {and to} \quad&{\textbf{x}}_l\le {\textbf{x}}\le {\textbf{x}}_u . \end{aligned}$$

This formulation, with *f* as the objective function, ${\textbf{x}}$ as the design variables (with ${\textbf{x}}_l$ and ${\textbf{x}}_u$ the lower and upper bounds), ${\textbf{y}}$ as the environmental and/or operational conditions, $g_i$ as inequality constraints, and $h_j$ as equality constraints, is favored for its ability to produce clear and concise results, making it highly suitable for demonstrating new SBDO methodologies in marine engineering.

Despite the potential to address a broader spectrum of design criteria, the uptake of multi-objective optimization, that reformulate the problem in Eq. 3 as follows

2 $$\begin{aligned} \min _{{\textbf{x}}} \quad&\{f_1({\textbf{x}},{\textbf{y}}), f_2({\textbf{x}},{\textbf{y}}), \ldots , f_k({\textbf{x}},{\textbf{y}})\}\nonumber \\ \text {subject to} \quad&g_i({\textbf{x}},{\textbf{y}}) \le 0, \quad i = 1, \ldots , m \nonumber \\ \text {and to} \quad&h_j({\textbf{x}},{\textbf{y}}) = 0, \quad j = 1, \ldots , p \nonumber \\ \text {and to} \quad&{\textbf{x}}_l\le {\textbf{x}}\le {\textbf{x}}_u, \end{aligned}$$

is cautious (see Fig. 5a, top). This approach, involving the simultaneous optimization of multiple conflicting *k* objectives, faces challenges due to its increase in required computational resources and complexity. Figure 6 presents a comprehensive depiction of the SBDO process using the extended design structure matrix (XDSM) [33]. This representation includes the three main blocks (shape parametrization, numerical solver, and optimizer) of the process, including also a stopping criteria, which may encompass either the convergence of the optimization method or constraints imposed by a limited computational budget.

The adoption of stochastic optimization (see Fig. 5a, bottom), which factors in uncertainty and variability, is still limited. Techniques like robust design optimization (RDO) [8, 26, 34–37], that focus on performance stability under uncertainty, reliability-based design optimization (RBDO) [24, 38–41], which emphasizes safety and reliability standards under probabilistic uncertainty models, and reliability-based and robust design optimization (RBRDO) [42–44], that combines RDO and RBDO approaches to ensure that a design is both robust against variability and reliable in terms of meeting safety or success criteria, are not yet widespread, pointing to a significant potential area of development in the field, representing only 9% of the existing literature.

Figure 5a clearly illustrates the continued preference for single-objective over multi-objective optimization (top) and deterministic over stochastic optimization (bottom) approaches in the marine engineering domain. These preferences underscore the field’s inclination towards methodologies that offer straightforward applicability and simplicity. Figure 5b, on the other hand, reveals a modest but growing interest in multi-objective optimization, with a limit to the number of objectives, indicating a cautious approach to embrace complexity in optimization challenges. Examples of many-objectives optimization problems (number of objectives greater than 3) are given in [45–49] for 4 objectives and in [50–52] for 5 objectives.

Furthermore, the analysis of problem formulations in SBDO studies, as depicted in Fig. 5c, reveals that a significant majority of problems (63%) are formulated with constraints. This indicates that complex real-world conditions and requirements are typically encountered in marine engineering applications. Constraints in SBDO may originate from design, regulatory and safety requirements, physical limitations, and environmental considerations.

The predominance of constrained problems underscores the need for optimization methodologies that can effectively account for these limitations, balancing the achievement of design objectives with adherence to constraint boundaries. Interestingly, a notable 19% of the problems are identified as unconstrained. This suggests scenarios where design freedom is less restricted, possibly in more theoretical or exploratory studies, or in cases where the primary focus is on optimizing a single aspect of design without the need for balancing it against other factors. Another possibility is the use of implicit geometrical constraints, such that they don’t need to be considered in the problem formulation anymore because they are satisfied by definition. However, Fig. 5c also highlights a critical gap in current SBDO research—a lack of clarity or information regarding the problem formulation in 18% of the papers. This ambiguity in the formulation, specifically the absence of clear statements on whether the problems are constrained or not, points to a potential oversight in the documentation or conceptualization of SBDO studies. It raises questions about the comprehensiveness and depth of problem understanding in these cases. The absence of explicit mention of constraints may lead to challenges in replicating or building upon the research, as the constraints (or lack thereof) significantly influence the optimization process and outcomes. Furthermore, the figure brings to light an important aspect of SBDO that appears to be insufficiently addressed: the strategies for dealing with constraints. Effective constraint handling is crucial in SBDO, as it directly impacts the feasibility and practicality of the optimized solutions. The lack of detailed discussion on constraint management techniques in a considerable number of studies suggests a need for more focused research in this area. This includes the development and application of advanced constraint-handling techniques, which are essential for ensuring that the solutions generated by SBDO are not only optimal in a mathematical sense but also viable and effective in real-world applications.

The scoping review has finally highlighted a notably sparse yet significant application of multidisciplinary design optimization (MDO) methodologies within the broader context of SBDO in marine engineering, encompassing only about 8% of the studies. This is particularly noteworthy in a field inherently requiring integration across various disciplines such as hydrodynamics, structural engineering, and materials science for optimal design solutions. MDO problems focusing on resistance/powering and seakeeping performance improvement have been addressed in the context of various vessels, including surface combatant [44, 53], frigate [54], and multi-hulls [43, 55]. These studies highlight the application of MDO in enhancing specific performance parameters of marine vehicles. A multilevel hierarchy system approach, which allows for the integration of results from synthesis-level optimization into subsystem optimization and overall coordination of multi-level design systems, was demonstrated in studies like [56] and [57]. These works employed methods like constructive artificial neural networks for the MDO of twin H-body vessels and multi-hulls, considering objectives and constraints related to cavitation, structural integrity, stability, hull forms, weights, costs, and payload capacity. System-level MDO, considering seakeeping, maneuvering, and resistance assessment, was explored in [45], showcasing the comprehensive nature of this MDO approach. In contrast, a generalized collaborative optimization (CO) method for resistance optimization of small water-plane area twin hull (SWATH) vessels was proposed in [58], signifying the adaptability of CO in focused optimization tasks. The optimization of an autonomous underwater vehicle (AUV) for various performance metrics such as rapidity, maneuverability, resistance, and energy consumption through CO was undertaken in studies like [59] and [60]. Additionally, a modified bi-level integrated system collaborative optimization for resistance and weight reduction of a SWATH was proposed in [61]. The application of a multi-objective MDO based on the all-at-once architecture for weight minimization and endurance maximization of an AUV was demonstrated in [62]. Resistance optimization and wake flow uniformity of an offshore aquaculture vessel were addressed in [63], while [64] utilized a concurrent subspace design method for comprehensive MDO of an AUV, covering hull form, structure, propulsion, energy, maneuverability, and general arrangement. Further studies explored a range of MDO applications [65], from hydrostructural optimization [9, 38, 66] to energy consumption minimization [67], showcasing the diversity of MDO applications in marine engineering, employing various architectural approaches such as fluid–structure interaction coupling [68], super element-based multi-level analysis [69], and uncertainty quantification in system-level MDO [70].

**Fig. 7:**
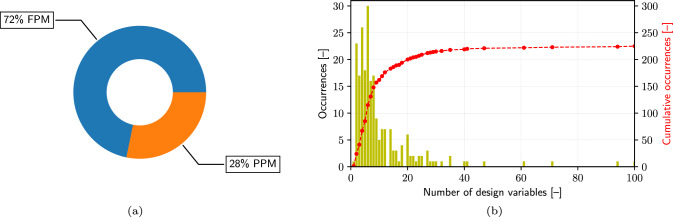
Occurrences of (**a**) fully- versus partially-parametric modeling for shape modification and (**b**) distribution of design-space dimensionality

### Design-Space Parameterization

In the realm of SBDO, the parametrization of the design space is a critical step that significantly influences the optimization process. Parametrization can be categorized broadly into fully-parametric (FPM) and partially-parametric models (PPM) [71]. FPMs define every aspect of the design using parameters, offering high control and predictability. PPMs, however, combine parametric elements with non-parametric or fixed aspects, providing a balance between control and flexibility. This distinction is crucial in SBDO, where the choice of parametrization technique impacts the feasibility, efficiency, and scope of the optimization task.

Figure 7a shows the predominant preference for FPM, accounting for 72%. This dominance suggests a trend towards well-defined, controlled, and interpretable approaches in design variable specification. FPM approaches include CAD-based [72], analytical [73, 74], scaling [57], sectional area curves [75–77], partial differential equations [1], Ferguson [40], Legendre [78], Bezier curves [17, 35, 79–82] and surfaces [3, 70], Splines [83, 84], B-splines [7, 51, 85–94], T-splines [95], F-splines [96], NURBS [6, 97–101], PARSEC [102], Lackeby [103, 104], and Akima [105]. On the other hand, PPM methods such as free-form deformation (FFD) [63, 66, 106–124], radial basis functions (RBF) [55, 125–130], arbitrary shape deformation [131–135], patches [136–139], blending [136, 140, 141], and morphing [142], accounting for 28%, are indicative of the need for more adaptable and flexible design approaches. Overall, Splines family (Spline, NURBS, B-Spline, T-Spline) approaches are the most used among the FPM, whereas FFD is the most used among the PPM methods.

Figure 7b illustrates the distribution of design space dimensionalities and the cumulative sum of the associated occurrences. Most studies concentrate on problems with 10 dimensions or fewer, indicating a focus on moderately complex design challenges. However, the presence of problems with higher dimensionality, greater than 50 [66, 97, 113, 143–145], up to 420 dimensions [146], reveals the presence of applications with highly complex and high-dimensional optimization challenges. These high-dimensional optimizations are often facilitated by the use of adjoint gradients [34, 66, 130, 146, 147], since the computational cost of adjoint gradients scales favorably with the number of problem dimensions. Despite this success, adjoint solvers are not commonly used in the maritime field. This could be due to the relatively high complexity of these solvers which hampers a widespread adoption of the adjoint method for high-dimensional problems. Because of its high potential, research on adjoints for optimization should receive more attention. It is finally important to note that a significant portion of the works reviewed, approximately 26%, do not explicitly specify the dimensionality of the design space. This omission indicates a gap in the reported information, meaning the presented distribution may only partially represent the problem dimensionalities encountered in SBDO research. The absence of detailed dimensionality data underscores a potential area for improvement in the clarity and completeness of reporting in the field.

The problem dimensionality diversity raises the issue of the *curse of dimensionality* [148], where larger design spaces exponentially increase computational costs and complicate the optimization process. Despite the variety of methods used for SBDO, considering both FPM and PPM, the definition of the design space still represents the true bottleneck in design processes. By limiting free variables, parametric models can significantly save time and costs. Hence, choosing restrictions based on experience, constraints from production, operational requirements, and market acceptance is crucial. Good parametric models stem from conscious choices of restriction, emphasizing the need for dimensionality reduction techniques in SBDO.

The development of dimensionality reduction techniques for shape optimization only recently gained attention. The simplest method to reduce the dimensionality of the design space is to identify the most important variables for the design problem and discard the remaining ones by setting them to a constant value during the optimization process, i.e. a factor screening, also known as feature selection. This process is conducted off-line (or upfront) the SBDO procedure. Sensitivity analysis has been used in [149] to prescribe the design space, whereas Pearson correlation coefficient has been used in [52] as a variable screening metric. On the contrary, online methods (during the SBDO procedure) have been proposed addressing dynamic space reduction in [129, 150], where not the dimensionality of the design space is assessed, but the design variable range, exploring roughly the whole design space at the beginning of the SBDO and then restricting the variables range runtime, focusing on the most interesting part of the domain. However, these approaches do not always provide the best solution, since factor screening is not able to evaluate the importance that the fixed variables could have during the optimization process, especially when combined with other variables, and dynamic space reduction could not take into account possible multi-modalities of the objective function, thus missing the optimum region. Hence, industrial design, in general, is increasingly searching for such dimensionality reduction methods that can capture, in a reduced-dimensionality space (possibly upfront), the underlying most promising directions of the original design space, preserving its relevant features and thereby enabling an efficient and effective optimization in the reduced space. The remedy has been found in dimensionality reduction techniques such as unsupervised learning, feature extraction, and modal representation, overall known as representation learning. These methods are capable of learning relevant hidden structures of the original design-space parameterization and have been developed focusing on the assessment of design-space variability and the subsequent dimensionality reduction before the optimization is performed. A method based on the Karhunen-Loève expansion (KLE, equivalent to the proper orthogonal decomposition, POD) has been formulated in [112] for the assessment of the shape modification variability and the definition of a reduced-dimensionality global model of the shape modification vector. No objective function evaluations nor gradients are required by the method. The KLE is applied to the continuous shape modification vector, requiring the solution of an eigenvalue problem for a Fredholm integral equation. The discretized Fredholm equation can be solved using principal component analysis. The method has been successfully applied to the optimization of the Delft catamaran in deterministic [151, 152] and stochastic [43, 153] conditions, the DTMB 5415 model [154], Wigley hull [155], as well as on different propellers [49, 92, 156]. Off-line methods improve shape optimization efficiency by reparameterization and dimensionality reduction, providing the assessment of the design space and the shape parameterization before optimization and/or performance analysis is carried out. The assessment is based on the geometric variability associated with the design space, making the method computationally very efficient and attractive (no simulations are required). Nevertheless, if the dimensionality reduction procedure is fed only with information on the shape modification vector, they may overlook the correlation between geometric variance and the actual objective function, since small variations in the geometry can produce significant variations in the objective function, e.g. flow separations and cavitation. For this reason, dimensionality reduction based on KLE has been extended to include physical information related to the optimization problem, resulting in significant improvements in both deterministic [157, 158] and stochastic [44] cases. A similar approach has been achieved via the active subspace method [122, 123], which involves the identification of the so-called active subspaces of the input parameter space by analyzing the sensitivity of the output with respect to the input parameters, often using gradient information. Obviously, the use of physical information has a computational cost and cannot always be afforded by designers upfront the SBDO procedure. For this reason, a further attractive proposal is to substitute physical information with physics-related geometrical parameters. A recent example has been provided in [159] where geometric moments are used to include physics information, applying it to two different ships.

### Numerical Solvers

**Fig. 8:**
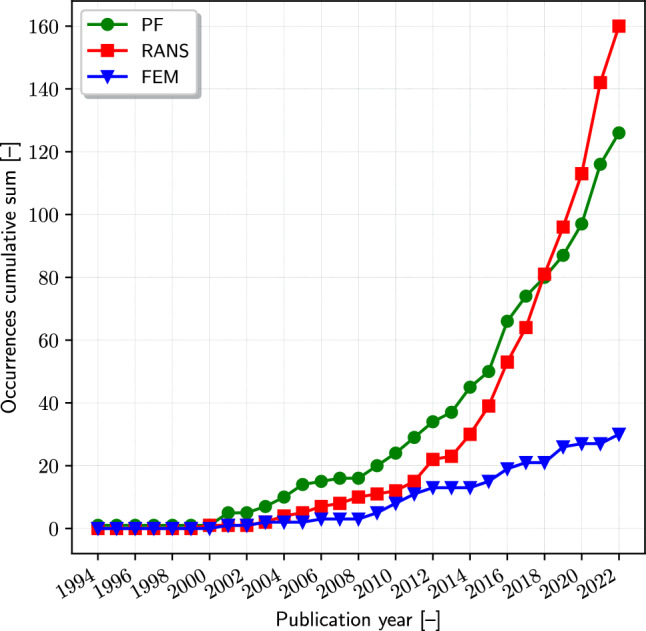
Cumulative sum of the kind of solvers used as a function of the publication year

Figure 8 presents a compelling overview of the evolving solver usage in SBDO studies from 1994 to 2022. The graph shows the cumulative sum of occurrences for various solvers. These are potential flow methods (PF), Reynolds-averaged Navier–Stokes (RANS), and the finite element method (FEM). Each solver represents distinct computational approaches in SBDO.

The PF solver, while exhibiting a consistent increase in cumulative occurrences over the years, has been outpaced by the RANS solver since 2018. The increase in PF usage indicates its continued relevance, particularly in problems where potential flow assumptions are valid, such as in the early stages of aerodynamic or hydrodynamic design. PF solvers are mainly based on the boundary elements method (BEM), see e.g. [24, 92, 160–164], but other examples have been found, such as strip theory [73, 91, 103, 165, 166], slender body [167], vortex lattice [4, 168], and blade element momentum [10, 46] methods, as well as isogeometric analysis combined with BEM [95, 159, 169]. It is important to recognize that within the realm of PF solvers, a significant portion are developed as proprietary, in-house tools, tailored to specific research or industrial needs. This trend underscores the specialized nature of PF solvers, which often require customization to address unique challenges in fluid dynamics and hydrodynamics. Nevertheless, commercially available options have also been used, see e.g. [25, 85, 96, 127, 128, 149, 170–174].

The RANS solver shows a quartic trend in its cumulative occurrences. This significant rise reflects the growing preference for RANS in SBDO studies. The main cause is likely due to its enhanced capability in capturing complex turbulent flows and its applicability in a broader range of fluid dynamics problems compared to PF. This, in combination with an increase of computational resources which makes RANS affordable for practical applications, results likely in a strong increase of RANS usage over the years. The quartic nature of the trend suggests an accelerating adoption rate, highlighting RANS as an increasingly preferred tool for fluid dynamics optimization in recent years, as also reflected by the distribution between commercial (see, e.g., [15, 16, 175–187]), in-house developed [188–190], and open-source [23, 191–197] solvers that is notably balanced. Commercial tools are widely used in various industries for their comprehensive capabilities and robust support structures. On the other hand, there are several notable in-house RANS solvers, which are developed within academic or research institutions for specific applications or research purposes.

Finally, the use of FEM solvers [116] shows a more limited cumulative occurrence in SBDO studies despite its critical role in structural analysis. This might be indicative of the specific focus of the studies under consideration, possibly skewed more towards fluid dynamics than structural optimization. However, the presence of FEM, mainly composed of commercial software, see e.g., [198–201], underscores its importance in the SBDO landscape, particularly for problems involving structural response and material optimization.

**Fig. 9:**
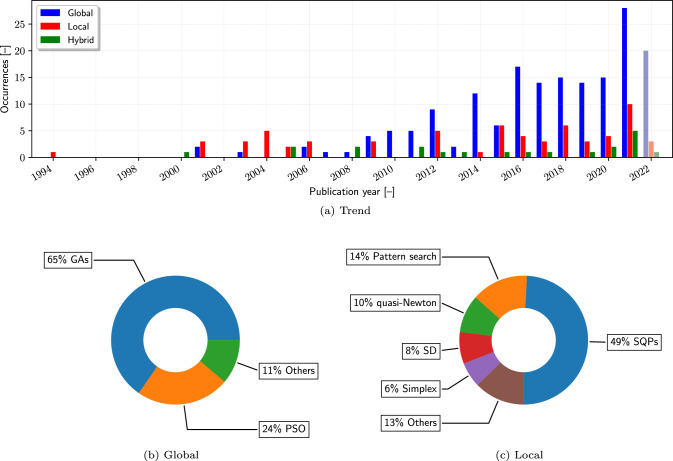
Optimization algorithm occurrences (**a**) trend and subdivision by (**b**) global and (**c**) local categories

The trends observed in Fig. 8 are indicative of the evolving preferences and technological advancements in the field of SBDO. The overtaking of PF by RANS in recent years points to a paradigm shift in solver selection, driven possibly by the increasing complexity of design problems and the need for more sophisticated fluid dynamics modeling capabilities. The limited but present use of FEM highlights the diverse range of optimization challenges addressed in SBDO, necessitating a variety of computational tools to cater to different aspects of marine engineering design.

### Optimization Methods

In the evolving landscape of SBDO, the selection of optimization algorithms and the possible integration of surrogate methods play pivotal roles. These strategies are key in navigating the complex design spaces and computational challenges inherent in SBDO. The choice between global, local, or hybrid algorithms, as well as the adoption of surrogate-based approaches versus surrogate-free methods, reflects a strategic balance between exploration and exploitation, accuracy, and computational efficiency.

#### Algorithms

Figure 9a illustrates the year-by-year usage of global, local, and hybrid algorithms in SBDO studies. The trend towards global optimization algorithms signifies a strategic shift in SBDO. Global algorithms, known for their ability to explore the entire design space, are increasingly favored. This preference likely stems from their stochastic nature and heuristic methods, which are adept at avoiding local optima: a critical advantage in complex, multimodal design landscapes. The rising trend of global algorithms suggests an industry-wide acknowledgment of the complexity and unpredictability inherent in SBDO problems.

Within the realm of global optimization, genetic algorithms (GAs, see, e.g., [11, 202–217]) and particle swarm optimization (PSO, see, e.g., [14, 218–221]) dominate. As shown in Fig. 9b, GAs cover 65% of global methods, leveraging mechanisms inspired by biological evolution, such as selection, crossover, and mutation. This allows for a robust exploration of the design space, making them particularly effective for non-linear, discrete, or mixed-variable optimization problems. PSO, with 24%, employs a swarm intelligence approach that simulates social behavior patterns, providing a balance between exploration and exploitation in the search process. Within the remaining 11% of the global methodologies, several notable algorithms have been identified and warrant mention. These include the infeasibility-driven evolutionary algorithm [87, 184, 222], simulated annealing [26, 87], artificial bee colony [126, 223], and dividing rectangles [154, 224].

**Fig. 10:**
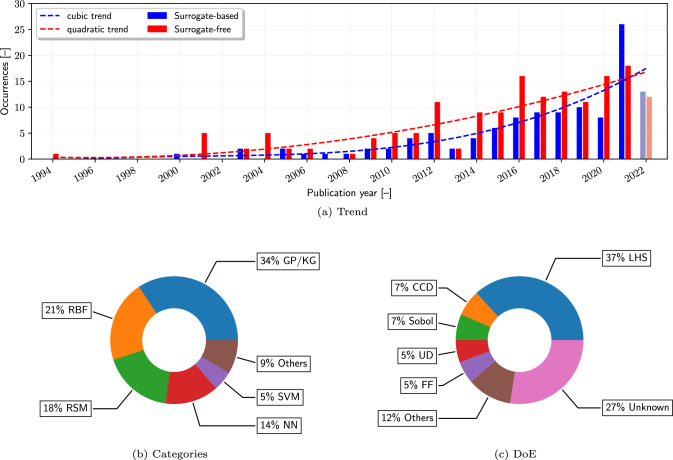
Surrogate-based versus surrogate-free occurrences trends (**a**), surrogates categories (**b**), and design of experiments used for initial training (**c**)

Considering local methods, the preference for sequential quadratic programming (SQP, see, e.g., [77, 84, 225–229]) and methods like quasi-Newton [1] methods (e.g., the Broyden-Fletcher-Goldfarb-Shanno, BFGS algorithm [91, 230]) and pattern search, also known as Hooke and Jeeves algorithm [103, 165, 182, 231], as seen in Fig. 9c, aligns with problems where a good initial guess is available, and the design space is less rugged. In particular, SQP, with its ability to handle nonlinear constraints efficiently, is apt for fine-tuning solutions within a well-defined local region, complementing the global search methodologies. The steepest descent (SD) algorithm [3], the simplex method, also known as Nelder-Mead algorithm [97, 127, 232, 233], and other gradient-based approaches [234] are overall less preferred.

Finally, hybrid approaches deserve some hints. It may be noted that hybrid approaches include both memetic approaches (hybrid global/local) [60, 110, 153, 158, 175, 176, 189, 235], as well as hybridization of different global algorithms [108], global methods with reinforcement learning [236], and local algorithms with multi-start approaches [164, 200]. Among the memetic approaches the SHERPA (simultaneous hybrid exploration that is robust, progressive, and adaptive) algorithm [18, 55, 134, 135, 237, 238], noted for its robust and adaptive capabilities in handling complex design challenges, is gaining recognition in various engineering domains, not only marine. However, its proprietary nature, being exclusive to a specific software environment, presents potential limitations in terms of widespread adoption and accessibility, particularly in academic and open-source research communities where transparency and adaptability of algorithms are often paramount.

#### Surrogates

Figure 10a compares the trend of solving SBDO problems with and without surrogate methods. The recent overtaking of surrogate-based methods over surrogate-free approaches marks a significant development in SBDO. In surrogate-based optimization, the original optimization problem in Eq. 3 is reformulated by approximating the objective function ${f}({\textbf{x}})$ and the eventual functional constraints ${g}_i({\textbf{x}})$ and $h_j({\textbf{x}})$ with surrogate models, denoted as $\hat{f}({\textbf{x}})$, $\hat{g}_i({\textbf{x}})$, and $\hat{h}_j({\textbf{x}})$ respectively. This approach transforms the original optimization task into a more computationally tractable form by minimizing the surrogate objective function while ensuring that surrogate constraints are satisfied. The reformulated optimization problem is expressed as:

3 $$\begin{aligned} \min _{{\textbf{x}}} \quad&\hat{f}({\textbf{x}},{\textbf{y}}) \nonumber \\ \text {subject to} \quad&\hat{g}_i({\textbf{x}},{\textbf{y}}) \le 0, \quad i = 1, \ldots , m \nonumber \\&\hat{h}_j({\textbf{x}},{\textbf{y}}) = 0, \quad j = 1, \ldots , p . \end{aligned}$$

Surrogate models, serving as approximations of the actual objective and constraint functions, offer substantial computational savings. The cubic trend of surrogate-based methods (see Fig. 10a) reflects their growing importance in dealing with high-fidelity simulations that are computationally expensive, allowing for more iterations and a deeper exploration within feasible turnaround times.

The predominance of Gaussian process (GP, see, e.g., [52, 91, 121, 156, 190, 194]) and Kriging (KG, see, e.g., [7, 26, 34, 45, 58, 108, 114, 193, 209, 239–246]) methods (34%) in surrogate-based optimization, as shown in Fig. 10b, underscores their efficacy in capturing complex, nonlinear relationships with a relatively small number of samples. When it comes to practical applications, the distinction between GP models and KG models can become blurred despite their differences in original contexts and typical interpretations. This is particularly true in the context of surrogate modeling. In many cases, especially in computer experiments and design of experiments, the terms are used interchangeably, as the underlying mathematical principles are very similar. Both methods are highly appreciated for their ability to provide accurate predictions (excelling in modeling smooth functions) and a statistical framework that quantifies prediction uncertainty which is crucial for decision-making in the optimization process. However, computational challenges occur when applied to large datasets. Other popular methods like RBF (21%, see, e.g., [50, 127, 134, 149, 151, 189, 223, 247, 248]), response surface methodologies (RSM, 18%, see, e.g., [15, 16, 39, 59, 106, 109, 140, 141, 180, 249–252]), neural networks (NN, 14%, see, e.g., [4, 17, 20, 56, 57, 86, 162, 173, 175, 197]), and support vector machines (SVM, 5%, see, e.g., [63, 104, 199, 253, 254]) each offer unique advantages, such as local approximation capabilities and flexibility in modeling complex patterns. Specifically, RBFs are beneficial for multidimensional interpolation and smooth transitions, though they can struggle with larger, high-dimensional data; RSM is effective for design of experiments and process optimization but is less suited for non-linear or complex problems and requires extensive experimentation for accurate modeling; NNs, with their flexibility for complex relationships, are ideal for large datasets, but require significant data and are computationally intensive; lastly, SVM provide robust performance in high-dimensional spaces but are sensitive to kernel and parameter choices and computationally demanding for large datasets. This nuanced understanding of each method’s strengths and weaknesses is crucial in guiding the selection of the most appropriate surrogate modeling technique for specific engineering optimization problems. Finally, the other 9% surrogate-based approaches are composed of trust-region methods [2, 53], elliptic basis function [255], orthogonal polynomial methods [256], and hyper-surrogate approaches [186], where multiple surrogate methods are used, like RSM, RBF, and KG, and then averaged to get the objective prediction.

It may be noted that in the present scoping review, and under the statistics provided in Fig. 10b, works that characterize RBF models as single-layer NN are categorized under the use of RBF surrogates, rather than as conventional NN implementations. This classification stems from the mathematical alignment of single-layer RBF networks with RBF interpolation, highlighting their role as surrogate modeling techniques. In these instances, the RBF’s function is used primarily to approximate complex nonlinear relationships within the data, distinguishing it from the multi-layered, deep-learning frameworks typically associated with NNs.

**Fig. 11:**
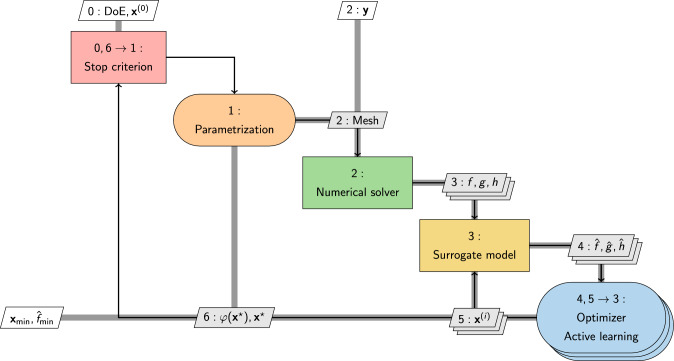
Example of extension of the XDSM diagram towards single-fidelity surrogate-based SBDO with active learning

Transitioning to another critical aspect of surrogate-based optimization, it is essential to acknowledge the pivotal role of the initial training and sampling approach employed for the surrogate models. The effectiveness of surrogate methods, as discussed earlier, hinges significantly on the quality and representativeness of the initial training data or design of experiments (DoE) used to construct these models. This data fundamentally influences the surrogate’s ability to accurately capture the underlying behavior of the objective function and constraints. Therefore, the selection of an appropriate DoE becomes a key determinant in the success of surrogate-based optimization processes. Among the various DoE employed (see Fig. 10c), the Latin hypercube sampling (LHS), see, e.g., [39, 56, 58] covers 37% of the cases (including optimal [26, 239] and universal [194] LHS) and this can be attributed to its effectiveness in generating well-distributed samples across the design space, ensuring a representative and unbiased training set for surrogate models. Other techniques include central composite design (CCD, 7%, e.g. [141, 157, 178, 180, 238], Sobol (7%, e.g. [120, 127, 250]), uniform design (UD, 5%, e.g. [48, 54, 199]), full factorial (FF, 5%, e.g. [229, 241, 257]), and finally the remaining 12% includes orthogonal arrays (5%, [50, 107, 189]), Hammersley/Halton sequences [70, 151, 156], as well as random/Monte Carlo sampling [37, 195, 197]. However, it is noteworthy that in 27% of the cases, the specific DoE strategy employed remains unidentified or unspecified. This lack of clarity on the training approach used can have implications for the interpretability and reproducibility of the optimization results. Consequently, this highlights a gap in the current body of research, underscoring the need for more transparent and detailed reporting of the sampling methodologies in surrogate-based optimization studies to better understand their impact on the effectiveness of the surrogate models.

In the domain of surrogate-based optimization, the development of multi-fidelity or variable-fidelity methods has emerged as a key strategy to enhance the effectiveness of surrogate models while also conserving computational resources. These methods leverage varying levels of model fidelity, combining computationally expensive high-fidelity simulations with less costly lower-fidelity approximations in order to construct more informed and efficient surrogates. Despite their potential benefits, the scoping review reveals that only 12% [2, 53, 61, 80, 91, 107, 142, 157, 189, 258] [230, 259, 260] of surrogate-based approaches have employed multi-fidelity methodologies, and their application appears sporadic over the years covered by the review. This limited utilization raises questions about the popularity and perceived benefits of multi-fidelity methods in this specific field. It is unclear whether this lack of widespread adoption is due to a general underutilization of these methods in the industry, or if there exist ambiguities and uncertainties regarding the actual advantages of integrating multi-fidelity approaches in surrogate-based optimization for marine engineering applications. This observation points to a potential area for further investigation and clarification, as the effective use of multi-fidelity methods could significantly impact the efficiency and accuracy of optimization processes in this domain.

In concluding the discussion on surrogate-based optimization, it is crucial to recognize the role of adaptive sampling or active learning methods in enhancing the effectiveness of these models 261. Such techniques, for both single- and multi-fidelity methods, start with an initial DoE, subsequently adapted by incorporating new samples ${\textbf{x}}^\star$ in areas most beneficial for optimization. A variety of strategies have been employed for this purpose, including, among others, the so-called acquisition function $\varphi$ based on: the validation of the best found [54, 64, 80, 86, 108, 151, 154, 180, 193, 244], the maximum uncertainty [107, 157], the expected improvement [34, 91, 157, 230], and lower confidence bounding [60, 157]. These methods aim to iteratively refine the surrogate model by focusing on regions of the design space where additional information can significantly influence the optimization outcome. Despite the apparent advantages of these adaptive techniques, this scoping review indicates that in 21% of the surrogate-based methods employing adaptive sampling approaches, the specific technique utilized remains unspecified. This lack of detail not only hinders the full understanding of the method’s implementation but also obscures the comparative analysis of different techniques’ efficacy. Given the potential impact of adaptive sampling on the accuracy and efficiency of surrogate-based optimization, particularly in marine engineering applications, this represents a significant gap in the current literature. A more transparent and detailed reporting of adaptive sampling methods could provide deeper insights into their benefits and limitations, fostering their more informed and effective use in the field.

An example of how SBDO workflow shown in Fig. 6 can be extended to the use of a general single-fidelity surrogate approach, including active learning, is given in Fig. 11. The diagram illustrates how the surrogate model acts as an intermediary between the numerical solver and the optimization algorithm. This arrangement facilitates the application of the optimization algorithm directly on the surrogate model to identify the optimal solution, denoted as ${\textbf{x}}_{\min }$ and $\widehat{f}_{\min }$. Concurrently, an active learning-driven optimization procedure operates in parallel. This procedure employs an acquisition function, $\varphi$, to systematically pinpoint potential new candidate solutions ${\textbf{x}}^{\star }$ to be sampled. These candidates are then processed through the numerical solver if the predefined stopping criterion has not yet been met. This dual-path approach integrates surrogate modeling with active learning to efficiently converge towards the optimum by balancing the exploration of the solution space and the exploitation of known high-potential areas. A further example of XDSM diagram extended to multi-fidelity methods can be found in [262].

**Fig. 12:**
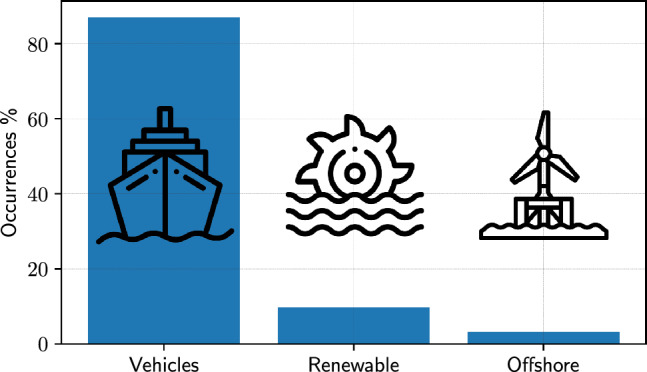
Occurrences of SBDO applied to marine engineering main applications fields

### Applications

Figure 12 shows the breakdown of the SBDO applications in marine engineering. The overwhelming majority of SBDO applications are dedicated to vehicle design (87%), which includes ships (see, e.g., [263–268]), submarines (see, e.g., [269, 270]), and various types of watercraft. This dominant focus can be attributed to several factors: (i) marine vehicles often have complex design requirements balancing hydrodynamic efficiency, stability, load capacity, and speed, consequently SBDO provides a powerful tool to optimize these competing factors; (ii) the marine vehicle industry is highly competitive, with a constant demand for improved performance and efficiency and SBDO enables designers to explore innovative shapes and configurations that might not be feasible through traditional design methods; (iii) the increasing environmental regulations and the push for energy efficiency drive the need for advanced optimization techniques to meet these stringent standards. The use of SBDO in the development of renewable energy solutions in marine settings, such as wave [22–25, 78, 186] and ocean-thermal [271] energy converters, pumps [272], and tidal [13, 14, 16–21], marine/ocean current [79, 81, 229, 241, 258, 273], river hydrokinetic [101, 163], and offshore wind [173, 182, 274] turbines, highlights its growing importance, covering 10% of the literature. This category’s smaller proportion might be due to the relatively newer field compared to traditional marine vehicle design. Furthermore, the design of renewable energy systems involves complex interactions with the marine environment, requiring sophisticated models that can be challenging to optimize. The smallest category in the breakdown is offshore applications (3%), which include steel catenary risers [39, 219], deep-sea test miners [239], platforms and semi-submersible structures [26, 247, 275], mooring systems [164], and ocean bottom flying nodes [257]. Factors influencing this lower percentage include high stakes and safety concerns, as well as complex environmental conditions. Offshore structures are often subject to stringent safety standards due to the high risks involved, possibly leading to a more conservative approach in adopting new optimization techniques. Moreover, the design of offshore structures must account for a wide range of environmental conditions, making the optimization process more challenging.

**Fig. 13:**
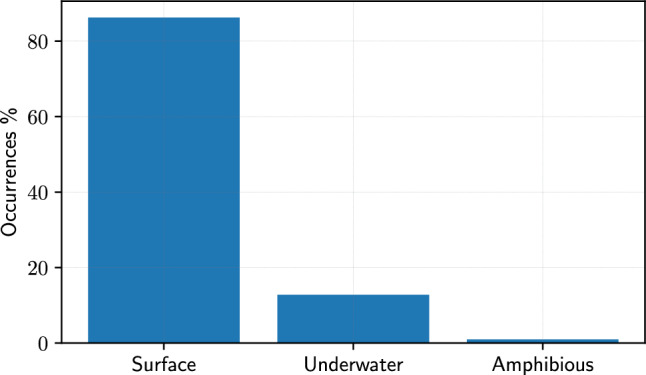
Occurrences of SBDO applied vehicles sub-categories

Among vehicle design, Fig. 13 offers insights into where optimization efforts are being primarily focused. Specifically, 86% is composed of surface vehicles, 13% underwater, and the remaining 1% amphibious. The optimization of surface vehicles can be pivotal in enhancing various aspects like hydrodynamic efficiency and seakeeping, resulting in less fuel consumption, improved stability and payload capacity. SBDO’s significant role in surface vehicle design may be due to the large economic and environmental impact of these vessels, driving a need for continuous improvement in their performance and efficiency. Underwater vehicles include submarines [72, 82] and autonomous underwater vehicles (AUVs, see, e.g., [178, 179, 183, 222]). The design optimization of these vehicles focuses on aspects like efficient maneuverability, stability under water, and energy efficiency for extended mission ranges. The application of SBDO in underwater vehicle design indicates a focus on specialized performance characteristics unique to the underwater environment, such as pressure resistance and stealth capabilities. Finally, amphibious vehicles [37, 65] are specialized vehicles that operate both in water and in air or land. The design challenges for amphibious vehicles are particularly complex due to the need to optimize performance in two very different environments. SBDO can play a key role in balancing these dual requirements, optimizing aspects such as buoyancy, stability, and propulsion efficiency.

**Fig. 14:**
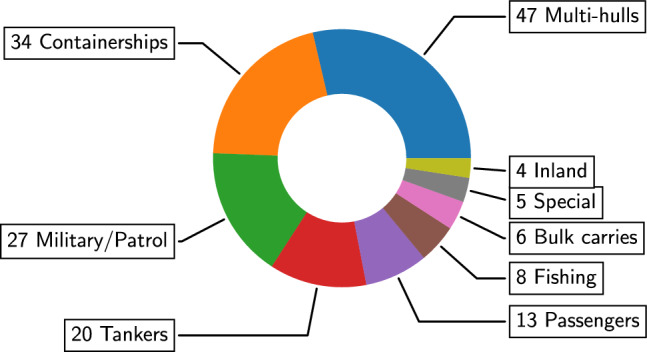
Occurrences of SBDO applied to surface vehicles sub-categories

Due to the predominance of surface vehicles, a further breakdown has been conducted in this subfield. The sub-categories are shown in Fig. 14. A significant focus on containerships (see, e.g., [234, 276]) in SBDO applications aligns with their vital role in global trade. Optimization for these vessels likely focuses on maximizing cargo capacity, fuel efficiency, and minimizing environmental impact, crucial for cost-effective and sustainable operations. The Korea research institute of ships and ocean engineering (KRISO) container ship (KCS) represents the most used benchmark in this sub-category, see, e.g., [6, 51, 77, 89, 110, 117, 122, 128, 180, 194, 234], serving as a standard reference model for various hydrodynamic studies. The optimization of military [54, 277, 278] and patrol [87, 231] vessels underscores the importance of performance, stealth, and agility in these applications. SBDO can be instrumental in enhancing these attributes, contributing to the effectiveness and safety of naval operations. As for containerships, also military vessels have their specific standard benchmark, represented by the David Taylor model basin (DTMB) 5415 model, which has been extensively used for hull-form optimization purposes [2, 44, 53, 109, 114, 131, 139, 145, 154, 158, 189, 224, 226, 260, 278]. The application of SBDO in tanker design (see, e.g., [278]) reflects the need for optimizing fuel efficiency and safety, given their role in transporting large volumes of liquid cargo, including oil and chemicals. The KRISO very large crude carrier (KVLCC2) model is the actual benchmark in this sub-category, see, e.g., [106, 245]. The application of SBDO in several further categories indicates a broad spectrum of optimization goals, from enhancing the efficiency of bulk carriers [111, 116, 214, 246] and fishing [47, 100, 115, 132, 140, 181, 232, 279] vessels to improving passenger comfort and safety in passenger’s vessels [99, 127, 150, 236], including yachts [1, 9, 36, 70, 175, 176, 192] and cruise ships [48, 121]. The optimization of inland [10, 94, 210, 225] and special ships also points to specialized requirements, perhaps related to shallow waters navigation or unique operational roles like research vessels [166, 280], survey ships [221], or offshore aquaculture [63, 119].

**Fig. 15:**
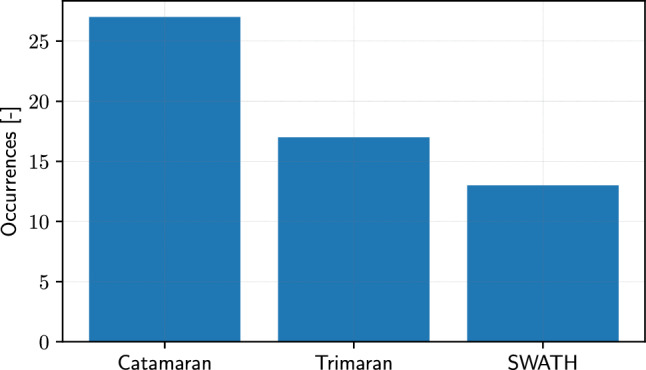
Occurrences of SBDO applied to multi-hulls sub-categories

As shown in Fig. 14, the strongest emphasis on surface vessels is represented by multi-hull designs, such as catamarans and trimarans, suggesting a focus on seakeeping and efficiency, resulting in improved stability and speed. Multi-hulls present unique design challenges that SBDO can help address, particularly in balancing stability with performance. For these reasons a deeper analysis has been conducted on multi-hull vessels, revealing three main sub-categories, which are catamarans, trimarans, and SWATH vehicles (see Fig. 15). Catamarans, with two parallel hulls of equal size, offer stability and spaciousness, making them popular for passenger ferries and recreational vessels. SBDO in catamaran design [85, 137] likely focuses on optimizing hull shape for stability [43, 107, 108] and reducing resistance, improving fuel efficiency [52, 127, 142, 162, 242, 282]. The standard benchmark model used for developing and assessing SBDO methodologies is represented by the Delft catamaran, see, e.g., [42, 112, 151, 152]. Trimarans, featuring a main hull with two smaller outrigger hulls, are known for their speed and stability, making them suitable for high-speed ferries and racing yachts. In trimaran design [57, 125], SBDO can play a crucial role in optimizing the hull configuration for balance and speed [141, 174, 211, 238], ensuring structural integrity [201] while maximizing performance [55, 104, 130, 134, 135, 147, 167, 283, 284]. The use of SBDO in trimarans can also address specific challenges like wave-piercing capabilities [254] and maneuverability, enhancing their performance in various marine conditions. SWATH vessels are designed to minimize hull volume at the water’s surface, reducing the impact of waves and providing a smoother ride in rough seas. SBDO in SWATH design [56, 58, 118, 161, 206, 230, 250, 252] is likely centered on optimizing the hull shape and configuration [61, 153, 249] to achieve the desired stability and seakeeping qualities [91, 190], making them ideal for applications like research vessels and coast guard ships. It should be finally highlighted that Fig. 14 does not account for the hull-form studies applied to the Wigley [97, 267] and systematic series S60 [83, 177, 202, 223, 263] benchmark models because they cannot be included in any of the specified subcategories. Nevertheless, they have been used for specific development/assessment of SBDO method [73, 74, 98, 124, 126, 129, 133, 155, 171, 172, 187, 220, 276, 285], as well as for particular operational/environmental conditions, like high speed [228] and shallow waters [137], or retrofitting [11, 243].

**Fig. 16:**
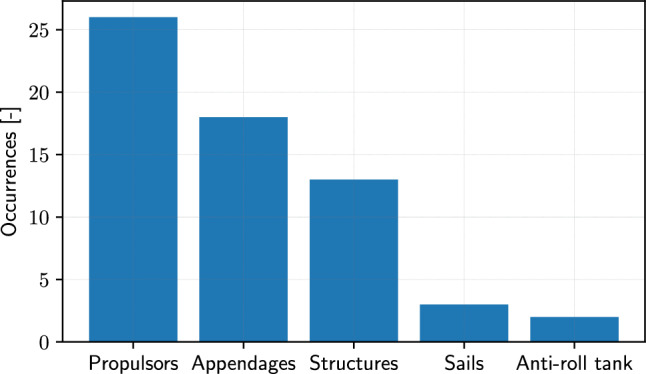
Occurrences of SBDO applied to marine components following the primary classification level

Finally, a breakdown of SBDO applied to marine components is presented in Fig. 16. Propulsors, including propellers [38], water jets [104, 240], and thrusters [253], are critical for the movement and maneuverability of marine vehicles. Shape optimization in this area focuses on improving hydrodynamic efficiency [5, 7, 46, 156, 185, 207, 259], reducing cavitation [8, 88, 92, 235], and minimizing noise [4, 168, 286]. The optimization could involve refining blade shapes and angles [12] to enhance propulsion efficiency while reducing fuel consumption [36, 49] and environmental impact [10, 11, 90], including also retrofitting solutions, like equalizing ducts [93]. Marine vehicle appendages include rudders [6], fins [66, 113], and keels [175], which play essential roles in stability and steering. Shape optimization in appendages [157, 169, 188, 191] aims to enhance hydrodynamic performance, improve maneuverability, and reduce drag [195]. This might involve optimizing the size, shape, and positioning of these components to achieve a balance between stability and agility [9, 70]. Structures likely encompass the hull and superstructure of marine vehicles, as well as substructures of offshore platforms [26, 173, 247, 272, 274]. Shape optimization in structures focuses on enhancing overall hydrodynamic performance, maximizing space utilization, and ensuring structural integrity [40, 200, 201, 287]. In addition, it involves tweaking hull forms for better wave resistance, stability, and seakeeping qualities, crucial for efficiency and safety [68, 203], including crashworthiness [143, 144, 198, 218]. In sailboats and sailing yachts, the optimization of sail shapes is vital for maximizing wind propulsion efficiency [244]. This involves determining the optimal curvature, material stiffness, and positioning of sails to harness wind power effectively, which is essential for performance in competitive sailing and leisure cruising [34, 102]. Finally, anti-roll tanks are used to stabilize ships by reducing rolling motion caused by waves. Shape optimization in anti-roll tanks aims to maximize their effectiveness in damping roll motion while minimizing the impact on the vessel’s overall performance and weight distribution [166, 245].

The detailed breakdown of shape optimization in various marine vehicle components underscores the comprehensive and multifaceted nature of design challenges in marine engineering. Shape optimization in each of these areas requires a deep understanding of fluid dynamics, material properties, and operational conditions. The focus on specific components like propulsors, appendages, and structures reflects the industry’s commitment to enhancing performance, safety, and environmental sustainability. The optimization of sails and anti-roll tanks highlights specialized areas where SBDO can significantly impact vessel performance and passenger comfort. This analysis demonstrates the critical role of shape optimization in advancing the design and functionality of marine vehicles. It highlights the technological advancements in SBDO and its application in addressing the nuanced and complex design requirements of different components of marine vehicles.

Overall, this analysis underscores the adaptability and potential of SBDO across various facets of marine engineering, promising continued innovation and improvement in the design of marine vehicles, renewable energy systems, and offshore structures.

## Discussion

The marine engineering field, while recognizing the advantages of more comprehensive multi-objective and stochastic optimization approaches, shows a marked preference for simpler, deterministic single-objective formulations. This trend results from the tendency to provide a simple and clear demonstration of new SBDO methodologies. At the same time, it highlights important areas for future growth such as the adoption of stochastic problem formulations, such as RDO, RBDO, and RBRDO. These approaches more accurately reflect the uncertainties characteristic of marine environments and align with broader trends in marine engineering, including digitalization, sustainability, and evolving regulatory landscapes. The analysis of problem formulations in SBDO studies reveals a landscape where constrained problems dominate, reflecting the complex nature of marine engineering challenges. However, the significant proportion of studies with unclear formulations and the apparent gap in the discussion of constraint-handling strategies highlight areas for improvement in SBDO research. Future studies would benefit from a more explicit focus on the nature and management of constraints, thereby enhancing the relevance, applicability, and impact of SBDO in marine engineering. The scarcity of MDO applications also highlights a potentially huge area for growth and development in marine engineering research. As the field continues to develop, an increased recognition of the benefits of a more integrated multidisciplinary approach is expected. MDO is especially useful in tackling complex design challenges that encompass multiple engineering facets. Future research could focus on developing more accessible and efficient MDO methodologies, facilitating their broader adoption in marine engineering optimization problems.

The variety of parameterization techniques reflects a range of approaches to defining design spaces, while the distribution of design space dimensionalities reveals both a focus on more manageable problems and an interest in tackling more complex, high-dimensional optimization challenges. This analysis underscores the need for continued innovation in SBDO methodologies, particularly in addressing the challenges posed by high-dimensional design spaces, and overcoming the curse of dimensionality. Dimension reduction techniques such as factor screening, sensitivity analysis, and dynamic space reduction are classical approaches to mitigate the curse of dimensionality. However, these techniques do not capture multi-modalities of the objective function and may therefore fail to find the optimum region. Unsupervised learning, feature extraction, and representation learning such as KLE and POD overcome these issues and do not require objective function evaluations or gradients. These methods are based on geometrical variance and do not account for the relation between geometrical variation and the variation of the objective. The inclusion of physical (objective) information is therefore identified as a promising way to improve dimension reduction techniques. Nevertheless, for practical application in an industrial context, where parametrization methods are mainly CAD-based, designers cannot easily retrieve the original design variables from the reduced design space (also known as the pre-image problem). It can be noted that a back-mapping procedure, called parametric model embedding (PME) [288], has been recently proposed. The PME simply extends the design-space dimensionality reduction procedure based on KLE/PCA using a generalized feature space that includes shape modification and design variables vectors together with a generalized inner product, building an embedded model of the original design parameterization.

The choice of numerical solvers in SBDO studies reflects an evolving landscape. The growing preference for RANS solvers over potential flow methods marks a shift towards more comprehensive fluid dynamics modeling. This transition aligns with the industry’s push towards capturing more complex, turbulent flows and the increasing availability of computational resources. However, the consistent but limited use of FEM solvers indicates a potential underutilization in structural optimization aspects of marine engineering. Future research could benefit from a more integrative approach that combines RANS for fluid dynamics with FEM for structural analysis, potentially leading to more comprehensive and effective optimization in marine engineering.

In the field of engineering optimization, the emphasis is often on achieving an optimal solution in a single iteration of an algorithm, reflecting the practical constraints of time and resources. Traditional stochastic global methods, while robust in exploratory capacity, typically require multiple iterations to ascertain solution reliability due to their inherent randomness. This necessitates a shift towards deterministic variants of global evolutionary strategies and population-based methods. These deterministic adaptations aim to retain the broad exploratory characteristics of global methods but enhance the consistency and predictability of outcomes in each individual run. Additionally, the strategic integration of these deterministic global methods with deterministic local search techniques marks a significant advancement in optimization practice. This hybrid approach synergistically merges the expansive exploration capabilities of global methods with the focused, efficient refinement of local optimization techniques, such as gradient-based or line search methods. The result is an approach that effectively leverages the strengths of both methodologies, facilitating convergence to the most optimal solution within the constraints of a single algorithmic execution. Such developments in deterministic global methods, complemented by hybridization with local searches, are particularly salient in engineering contexts. They offer a streamlined and effective means of identifying the global optimum, aligning with the practical exigencies of engineering optimization where timely and reliable solutions are paramount.

The trends and preferences in optimization algorithms and surrogate methods in SBDO reflect an evolving field that continually adapts to the intricacies of marine engineering design problems. The shift towards global optimization and the increasing reliance on surrogate-based methods indicate a strategic response to the challenges of high-dimensional, complex design spaces. This evolution underscores the industry’s commitment to finding a balance between computational efficiency and the need for thorough, accurate design exploration. It can be noted how the extension to multi-fidelity approaches, as well as, the integration of active learning/adaptive sampling procedure for the surrogate training process, is however still limited. These two branches represent a pathway to follow for future research to assess clearly the pros and cons of multi-fidelity versus single-fidelity methods, as well as identify the most efficient and effective DoE in combination with active learning/adaptive sampling procedure. It may be emphasized that, as for the problem formulation, the literature presents several unclear statements on which DoE is used for surrogate training, as well as what kind of acquisition function has been used in the case of active learning. This represents a huge gap in interpretability and repeatability of the methodologies, that have to be filled.

Finally, the current distribution of SBDO applications in marine engineering indicates a strong focus on vehicle design, reflecting both the industry’s needs and the maturity of optimization techniques in this area. However, the presence of renewable energy and offshore applications, although smaller in proportion, is significant. It suggests a growing recognition of SBDO’s potential in these areas, particularly in response to global trends toward sustainable energy and the need for environmentally resilient offshore infrastructure. As the field of SBDO evolves, it might be expected to see a diversification in its applications. The renewable energy sector, in particular, may experience growth in SBDO applications as the demand for sustainable energy solutions increases. Furthermore, advancements in SBDO methodologies might lead to greater adoption in offshore applications, addressing the unique challenges posed by these environments. The distribution of SBDO applications across different types of marine vehicles reflects the diverse challenges and priorities in marine vehicle design. The prominence of SBDO in surface vehicle optimization aligns with the global scale and economic significance of these vessels. The focus on underwater vehicles highlights the technological advancements and specialized requirements in this sector. Meanwhile, the application in amphibious vehicle design, although likely less in comparison, underscores the complexity and innovation in multi-environment vehicle design. SBDO is a crucial tool in advancing the design and performance of various types of marine vehicles, addressing unique challenges, and contributing to the evolution of more efficient, capable, and environmentally friendly marine transportation and exploration technologies. The breakdown of SBDO applications across various types of surface ships demonstrates the versatility and significance of optimization techniques in addressing the diverse design and operational challenges of different ship categories. The focus on containerships and military vessels reflects economic and strategic priorities, while the emphasis on multi-hulls indicates an interest in innovative hull designs. The diverse application across other ship types, such as tankers, bulk carriers, fishing, and passenger ships, highlights the broad applicability of SBDO in enhancing various aspects of marine vessel design and operation.

In summary, while SBDO has become a cornerstone in marine engineering, there is a clear path forward for further advancements. Embracing complex optimization methodologies, expanding the use of MDO, and integrating various computational solvers could pave the way for more innovative and sustainable solutions in marine engineering. These developments, coupled with the broader trends in digitalization and environmental consciousness, are poised to significantly shape the future of SBDO in this field.

It finally should be noted that although focusing exclusively on peer-reviewed journal papers has ensured the academic rigor and reliability of the sources reviewed, it may have limited the representation of industrial applications of SBDO in marine engineering. Industrial projects, especially those involving multi-objective and constrained optimization problems as well as multi-disciplinary efforts, are often not documented in the academic databases surveyed. This is due to various factors, including proprietary considerations and the publication venues typically preferred by industry practitioners, such as industry magazines, conference contributions, and books detailing larger research and development projects.

## Conclusions

The scoping review conducted in this study underscores the increasingly pivotal role of simulation-based design optimization (SBDO) in marine engineering. Our findings illuminate how SBDO is not just a facilitator of improved performance and cost-efficiency in marine engineering systems and components but also a catalyst for innovation and adaptation in the face of evolving technological and environmental challenges.

Significantly, our analysis reveals a low use of more sophisticated, multi-objective, and stochastic optimization approaches in SBDO, despite the complex, dynamic nature of marine environments. There remains a predominant reliance on simpler, deterministic single-objective formulations, highlighting a crucial area for future development. This gap underscores the necessity for more advanced algorithms that can more accurately model and navigate the uncertainties inherent in marine engineering, including factors like wave dynamics and ocean currents.

Moreover, the review highlights the emergence of high-fidelity solvers in SBDO, signaling a shift towards more nuanced and detailed simulation capabilities. This advancement is indicative of the field’s progression towards tackling more complex optimization challenges, further driven by the integration of active learning and adaptive sampling techniques in surrogate-based optimization models and the development of design-space dimensionality reduction procedures for addressing the curse of dimensionality problem.

In conclusion, this scoping review not only reaffirms the significant potential of SBDO in revolutionizing marine engineering practices but also identifies critical pathways for future research. These include the need for more integrative, multidisciplinary approaches, and the development of optimization methods that are both computationally efficient and robust in the face of the unique challenges posed by the marine environment. As the field continues to evolve, these insights will be instrumental in guiding the next generation of research and innovation in SBDO, paving the way for more sustainable, efficient, and advanced marine engineering solutions.

## References

[1] Lowe TW, Bloor MI, Wilson MJ (1994) The automatic functional design of hull surface geometry. J Ship Res 38(4):319–328

[2] Campana EF, Peri D, Tahara Y, Stern F (2006) Shape optimization in ship hydrodynamics using computational fluid dynamics. Comput Methods Appl Mech Eng 196(1–3):634–651. 10.1016/j.cma.2006.06.003

[3] Peri D, Rossetti M, Campana EF (2001) Design optimization of ship hulls via CFD techniques. J Ship Res 45(02):140–149. 10.5957/jsr.2001.45.2.140

[4] Vesting F, Bensow RE (2014) On surrogate methods in propeller optimisation. Ocean Eng 88:214–227. 10.1016/j.oceaneng.2014.06.024

[5] Ma C, Cai H-P, Qian Z-F, Chen K (2014) The design of propeller and propeller boss cap fins (PBCF) by an integrative method. J Hydrodyn 26(4):586–593. 10.1016/S1001-6058(14)60066-4

[6] Chen C-W, Lin T-Y, Chen B-Y, Kouh J-S (2018) Parametric design and optimization of a pivoting s-type rudder for containerships. J Marine Sci Technol 26(6):1. 10.6119/JMST.201812_26(6).0001

[7] Nouri NM, Mohammadi S, Zarezadeh M (2018) Optimization of a marine contra-rotating propellers set. Ocean Eng 167:397–404. 10.1016/j.oceaneng.2018.05.067

[8] Mirjalili S, Lewis A, Dong JS (2018) Confidence-based robust optimisation using multi-objective meta-heuristics. Swarm Evolut Comput 43:109–126. 10.1016/j.swevo.2018.04.002

[9] Diez M, Peri D, Fasano G, Campana EF (2012) Hydroelastic optimization of a keel fin of a sailing boat: a multidisciplinary robust formulation for ship design. Struct Multidisc Optim 46(4):613–625. 10.1007/s00158-012-0783-7

[10] Favacho BI, Vaz JRP, Mesquita ALA, Lopes F, Moreira ALS, Soeiro NS, Rocha OFLd (2016) Contribution to the marine propeller hydrodynamic design for small boats in the Amazon region. Acta Amazon 46:37–46. 10.1590/1809-4392201501723

[11] Esmailian E, Ghassemi H, Zakerdoost H (2017) Systematic probabilistic design methodology for simultaneously optimizing the ship hull-propeller system. Int J Naval Architect Ocean Eng 9(3):246–255. 10.1016/j.ijnaoe.2016.06.007

[12] Lu Y, Wu C, Liu S, Gu Z, Shao W, Li C (2021) Research on optimization of parametric propeller based on anti-icing performance and simulation of cutting state of ice propeller. J Marine Sci Eng 9(11):1247. 10.3390/jmse9111247

[13] Kinnas SA, Xu W, Yu Y-H, He L (2012) Computational Methods for the design and prediction of performance of Tidal Turbines. J Offshore Mech Arctic Eng 134(1):011101. 10.1115/1.4003390

[14] Zhang D-S, Chen J, Shi W-D, Shi L, Geng L-L (2016) Optimization of hydrofoil for tidal current turbine based on particle swarm optimization and computational fluid dynamic method. Thermal Sci 20(3):907–912. 10.2298/TSCI1603907Z

[15] Shi W, Wang D, Atlar M, Guo B, Seo K-c (2015) Optimal design of a thin-wall diffuser for performance improvement of a tidal energy system for an AUV. Ocean Eng 108:1–9. 10.1016/j.oceaneng.2015.07.064

[16] Huang B, Usui Y, Takaki K, Kanemoto T (2016) Optimization of blade setting angles of a counter-rotating type horizontal-axis tidal turbine using response surface methodology and experimental validation: optimization of a counter-rotating type tidal turbine. Int J Energy Res 40(5):610–617. 10.1002/er.3383

[17] Sun Z, Li Z, Fan M, Wang M, Zhang L (2019) Prediction and multi-objective optimization of tidal current turbines considering cavitation based on GA-ANN methods. Energy Sci Eng 7(5):1896–1912. 10.1002/ese3.399

[18] Im H, Hwang T, Kim B (2020) Duct and blade design for small-scale floating tidal current turbine development and CFD-based analysis of power performance. J Mech Sci Technol 34(4):1591–1602. 10.1007/s12206-020-0321-2

[19] Khanjanpour MH, Javadi AA (2020) Optimization of the hydrodynamic performance of a vertical Axis tidal (VAT) turbine using CFD-Taguchi approach. Energy Conv Manag 222:113235. 10.1016/j.enconman.2020.113235

[20] Ambarita EE, Harinaldi I (2021) Nasruddin: computational study on multi-objective optimization of the diffuser augmented horizontal axis tidal turbine. J Marine Sci Technol 26(4):1237–1250. 10.1007/s00773-021-00812-2

[21] Yeo EJ, Kennedy DM, O’Rourke F (2022) Tidal current turbine blade optimisation with improved blade element momentum theory and a non-dominated sorting genetic algorithm. Energy 250:123720. 10.1016/j.energy.2022.123720

[22] Silva SR, Gomes R, Falcao A (2016) Hydrodynamic optimization of the UGEN: wave energy converter with U-shaped interior oscillating water column. Int J Marine Energy 15:112–126. 10.1016/j.ijome.2016.04.013

[23] Simonetti I, Cappietti L, Elsafti H, Oumeraci H (2017) Optimization of the geometry and the turbine induced damping for fixed detached and asymmetric OWC devices: a numerical study. Energy 139:1197–1209. 10.1016/j.energy.2017.08.033

[24] Tao J, Cao F, Dong X, Li D, Shi H (2021) Optimized design of 3-DOF buoy wave energy converters under a specified wave energy spectrum. Appl Ocean Res 116:102885. 10.1016/j.apor.2021.102885

[25] Bao X, Xiao W, Li S, Iglesias G (2021) Parametric study and optimization of a two-body wave energy converter. IET Renew Power Generat 15(14):3319–3330. 10.1049/rpg2.12254

[26] Yang H, Zhu Y (2015) Robust design optimization of supporting structure of offshore wind turbine. J Marine Sci Technol 20(4):689–702. 10.1007/s00773-015-0323-4

[27] Brereton P, Kitchenham BA, Budgen D, Turner M, Khalil M (2007) Lessons from applying the systematic literature review process within the software engineering domain. J Syst Softw 80(4):571–583. 10.1016/j.jss.2006.07.009

[28] Arksey H, O’Malley L (2005) Scoping studies: towards a methodological framework. Int J Social Res Methodol 8(1):19–32. 10.1080/1364557032000119616

[29] Peters MD, Godfrey CM, Khalil H, McInerney P, Parker D, Soares CB (2015) Guidance for conducting systematic scoping reviews. JBI Evid Implement 13(3):141–146. 10.1097/XEB.0000000000000050 10.1097/XEB.0000000000000050 26134548

[30] Munn Z, Pollock D, Khalil H, Alexander L, Mclnerney P, Godfrey CM, Peters M, Tricco AC (2022) What are scoping reviews? providing a formal definition of scoping reviews as a type of evidence synthesis. JBI Evid Synth 20(4):950–952. 10.11124/JBIES-21-00483 35249995 10.11124/JBIES-21-00483

[31] Sharma P, Goyal N (2023) How to write a scoping review? Int J Adv Med Health Res 10(1):53–56. 10.4103/ijamr.ijamr_91_23

[32] Tricco AC, Lillie E, Zarin W, O’Brien KK, Colquhoun H, Levac D, Moher D, Peters MD, Horsley T, Weeks L et al (2018) Prisma extension for scoping reviews (prisma-scr): checklist and explanation. Ann Internal Med 169(7):467–473. 10.7326/M18-0850 30178033 10.7326/M18-0850

[33] Lambe AB, Martins JRRA (2012) Extensions to the design structure matrix for the description of multidisciplinary design, analysis, and optimization processes. Struct Multidisc Optim 46:273–284. 10.1007/s00158-012-0763-y

[34] Lee H, Jo Y, Lee D-J, Choi S (2016) Surrogate model based design optimization of multiple wing sails considering flow interaction effect. Ocean Eng 121:422–436. 10.1016/j.oceaneng.2016.05.051

[35] Yin XB, Lu Y, Zou J, Wan L (2019) Numerical and experimental study on hydrodynamic bulbous bow hull-form optimization for various service conditions due to slow steaming of container vessel. Proc Instit Mech Eng M: J Enge Maritime Environ 233(4):1103–1122. 10.1177/1475090218811782

[36] Bacciaglia A, Ceruti A, Liverani A (2021) Controllable pitch propeller optimization through meta-heuristic algorithm. Eng Comput 37(3):2257–2271. 10.1007/s00366-020-00938-8

[37] Du Z, Mu X, Zhu H, Han M (2022) Identification of critical parameters influencing resistance performance of amphibious vehicles based on a SM-SA method. Ocean Eng 258:111770. 10.1016/j.oceaneng.2022.111770

[38] Young YL, Baker JW, Motley MR (2010) Reliability-based design and optimization of adaptive marine structures. Compos Struct 92(2):244–253. 10.1016/j.compstruct.2009.07.024

[39] Yang HZ, Zheng W (2011) Metamodel approach for reliability-based design optimization of a steel catenary riser. J Marine Sci Technol 16(2):202–213. 10.1007/s00773-011-0121-6

[40] Choi M-J, Cho H, Choi KK, Cho S (2015) Sampling-based RBDO of ship hull structures considering thermo-elasto-plastic residual deformation. Mech Design Struct Mach 43(2):183–208. 10.1080/15397734.2014.940463

[41] Hou YH, Liang X, Mu XY (2018) Hull lines reliability-based optimisation design for minimum EEDI. Brodogradnja 69(2):17–33. 10.21278/brod69202

[42] Pellegrini R, Serani A, Leotardi C, Iemma U, Campana EF, Diez M (2017) Formulation and parameter selection of multi-objective deterministic particle swarm for simulation-based optimization. Appl Soft Comput 58:714–731. 10.1016/j.asoc.2017.05.013

[43] Diez M, Campana EF, Stern F (2018) Stochastic optimization methods for ship resistance and operational efficiency via CFD. Struct Multidisc Optim 57(2):735–758. 10.1007/s00158-017-1775-4

[44] Serani A, Stern F, Campana EF, Diez M (2022) Hull-form stochastic optimization via computational-cost reduction methods. Eng Comput 38(S3):2245–2269. 10.1007/s00366-021-01375-x

[45] He J, Hannapel S, Singer D, Vlahopoulos N (2011) Multidisciplinary design optimisation of a ship hull using metamodels. Ship Technol Res 58(3):156–166. 10.1179/str.2011.58.3.004

[46] Kamarlouei M, Ghassemi H, Aslansefat K, Nematy D (2014) Multi-objective evolutionary optimization technique applied to propeller design. Acta Polytech Hungarica 11(9):163–182

[47] Lin Y, He J, Li K (2018) Hull form design optimization of twin-skeg fishing vessel for minimum resistance based on surrogate model. Adv Eng Softw 123:38–50. 10.1016/j.advengsoft.2018.05.010

[48] Wang P, Wang F, Chen Z, Dai Y (2021) Aerodynamic optimization of a luxury cruise ship based on a many-objective optimization system. Ocean Eng 236:109438. 10.1016/j.oceaneng.2021.109438

[49] Doijode PS, Hickel S, Van Terwisga T, Visser K (2022) A machine learning approach for propeller design and optimization: Part II. Appl Ocean Res 124:103174. 10.1016/j.apor.2022.103174

[50] Peri D, Campana EF (2005) High-Fidelity models and multiobjective global optimization algorithms in simulation-based design. J Ship Res 49(03):159–175. 10.5957/jsr.2005.49.3.159

[51] Lu Y, Chang X, Yin X, Li Z (2019) Hydrodynamic design study on ship bow and stern hull form synchronous optimization covering whole speeds range. Math Problems Eng 2019:1–19. 10.1155/2019/2356369

[52] Mittendorf M, Papanikolaou AD (2021) Hydrodynamic hull form optimization of fast catamarans using surrogate models. Ship Technol Res 68(1):14–26. 10.1080/09377255.2020.1802165

[53] Peri D, Campana EF (2003) Multidisciplinary design optimization of a naval surface combatant. J Ship Res 47(01):1–12. 10.5957/jsr.2003.47.1.1

[54] Peri D, Campana EF, Dattola R (2005) Multidisciplinary design optimization of a frigate. Ship Technol Res 52(4):151–158. 10.1179/str.2005.52.4.002

[55] Nazemian A, Ghadimi P (2021) Multi-objective optimization of trimaran sidehull arrangement via surrogate-based approach for reducing resistance and improving the seakeeping performance. Proc Instit Mech Eng M: J Eng Maritime Environ 235(4):944–956. 10.1177/1475090220980275

[56] Besnard E, Schmitz A, Hefazi H, Shinde R (2007) Constructive neural networks and their application to ship multidisciplinary design optimization. J Ship Res 51(04):297–312. 10.5957/jsr.2007.51.4.297

[57] Hefazi H, Mizine I, Schmitz A, Klomparens S, Wiley S (2010) Multidisciplinary synthesis optimization process in multihull ship design: multidisciplinary synthesis optimization process. Naval Eng J 122(3):29–47. 10.1111/j.1559-3584.2010.00240.x

[58] Xiao M, Gao L, Shao X, Qiu H, Jiang P (2012) A generalised collaborative optimisation method and its combination with kriging metamodels for engineering design. J Eng Design 23(5):379–399. 10.1080/09544828.2011.595706

[59] Luo W, Lyu W (2015) An application of multidisciplinary design optimization to the hydrodynamic performances of underwater robots. Ocean Eng 104:686–697. 10.1016/j.oceaneng.2015.06.011

[60] Luo W, Guo X, Dai J, Rao T (2021) Hull optimization of an underwater vehicle based on dynamic surrogate model. Ocean Eng 230:109050. 10.1016/j.oceaneng.2021.109050

[61] Jiang P, Zhou Q, Shao X, Long R, Zhou H (2016) A modified BLISCO method and its combination with variable fidelity metamodel for engineering design. Eng Comput 33(5):1353–1377. 10.1108/EC-06-2015-0164

[62] Liu X, Yuan Q, Zhao M, Cui W, Ge T (2017) Multiple objective multidisciplinary design optimization of heavier-than-water underwater vehicle using CFD and approximation model. J Marine Sci Technol 22(1):135–148. 10.1007/s00773-016-0399-5

[63] Feng Y, Chen Z, Dai Y, Wang F, Cai J, Shen Z (2018) Multidisciplinary optimization of an offshore aquaculture vessel hull form based on the support vector regression surrogate model. Ocean Eng 166:145–158. 10.1016/j.oceaneng.2018.07.062

[64] Zhang T, Zhou H, Wang J, Liu Z, Xin J, Pang Y (2019) Optimum design of a small intelligent ocean exploration underwater vehicle. Ocean Eng 184:40–58. 10.1016/j.oceaneng.2019.05.015

[65] Seth A, Liem RP (2020) Amphibious aircraft developments: computational studies of hydrofoil design for improvements in water-takeoffs. Aerospace 8(1):10. 10.3390/aerospace8010010

[66] Garg N, Kenway GKW, Martins JRRA, Young YL (2017) High-fidelity multipoint hydrostructural optimization of a 3-D hydrofoil. J Fluids Struct 71:15–39. 10.1016/j.jfluidstructs.2017.02.001

[67] Chen X, Wang P, Zhang D, Dong H (2018) Gradient-based multidisciplinary design optimization of an autonomous underwater vehicle. Appl Ocean Res 80:101–111. 10.1016/j.apor.2018.08.006

[68] Diez M, Lee EJ, Harrison EL, Powers AMR, Snyder LA, Jiang MJ, Bay RJ, Lewis RR, Kubina ER, Mucha P, Stern F (2022) Experimental and computational fluid-structure interaction analysis and optimization of deep-V planing-hull grillage panels subject to slamming loads - Part I: regular waves. Marine Struct 85:103256. 10.1016/j.marstruc.2022.103256

[69] Sun L, Wang D (2011) A new rational-based optimal design strategy of ship structure based on multi-level analysis and super-element modeling method. J Marine Sci Appl 10(3):272–280. 10.1007/s11804-011-1069-y

[70] Leotardi C, Serani A, Iemma U, Campana EF, Diez M (2016) A variable-accuracy metamodel-based architecture for global MDO under uncertainty. Struct Multidisc Optim 54(3):573–593. 10.1007/s00158-016-1423-4

[71] Harries S, Abt C (2019) Faster turn-around times for the design and optimization of functional surfaces. Ocean Eng 193:106470. 10.1016/j.oceaneng.2019.106470

[72] Vasudev KL, Sharma R, Bhattacharyya SK (2016) A modular and integrated optimisation model for underwater vehicles. Defence Sci J 66(1):71. 10.14429/dsj.66.8889

[73] Bagheri H, Ghassemi H (2014) Genetic algorithm applied to optimization of the ship hull form with respect to seakeeping performance. Trans FAMENA 38(3):45–58

[74] Bagheri H, Ghassemi H, Dehghanian A (2014) Optimizing the seakeeping performance of ship hull forms using genetic algorithm. TransNav Int J Marine Navig Saf Sea Transp 8(1):49–57. 10.12716/1001.08.01.06

[75] Park J-H, Choi J-E, Chun H-H (2015) Hull-form optimization of KSUEZMAX to enhance resistance performance. Int J Naval Architect Ocean Eng 7(1):100–114. 10.1515/ijnaoe-2015-0008

[76] Kim H-J, Choi J-E, Chun H-H (2016) Hull-form optimization using parametric modification functions and particle swarm optimization. J Marine Sci Technol 21(1):129–144. 10.1007/s00773-015-0337-y

[77] Park S-W, Kim S-H, Kim Y-I, Lee I (2022) Hull form optimization study based on multiple parametric modification curves and free surface reynolds-averaged Navier-stokes (RANS) solver. Appl Sci 12(5):2428. 10.3390/app12052428

[78] Coiro DP, Daniele E, Della Vecchia P (2016) Diffuser shape optimization for GEM, a tethered system based on two horizontal axis hydro turbines. Int J Marine Energy 13:169–179. 10.1016/j.ijome.2015.08.002

[79] Yang B, Shu XW (2012) Hydrofoil optimization and experimental validation in helical vertical axis turbine for power generation from marine current. Ocean Eng 42:35–46. 10.1016/j.oceaneng.2012.01.004

[80] Koziel S, Leifsson L (2012) Simulation-driven design using surrogate-based optimization and variable-resolution computational fluid dynamic models. J Comput Methods Sci Eng 12(1–2):75–98. 10.3233/JCM-2012-0405

[81] Luo XQ, Zhu GJ, Feng JJ (2014) Multi-point design optimization of hydrofoil for marine current turbine. J Hydrodyn 26(5):807–817. 10.1016/S1001-6058(14)60089-5

[82] Chrismianto D, Zakki AF, Arswendo B, Kim DJ (2015) Development of cubic Bezier curve and curve-plane intersection method for parametric submarine hull form design to optimize hull resistance using CFD. J Marine Sci Appl 14(4):399–405. 10.1007/s11804-015-1324-8

[83] Dejhalla R, Vukovic S, Mrsa Z (2001) Numerical optimisation of the ship hull from a hydrodynamic standpoint. Brodogradnja 49(3):289–294

[84] Suzuki K, Kai H, Kashiwabara S (2005) Studies on the optimization of stern hull form based on a potential flow solver. J Marine Sci Technol 10(2):61–69. 10.1007/s00773-005-0198-x

[85] Chen PF, Huang CH (2004) An inverse hull design approach in minimizing the ship wave. Ocean Eng 31(13):1683–1712. 10.1016/j.oceaneng.2003.08.010

[86] Grigoropoulos GJ, Chalkias DS (2010) Hull-form optimization in calm and rough water. Comput Aided Design 42(11):977–984. 10.1016/j.cad.2009.11.004

[87] Mohamad Ayob AF, Ray T, Smith WF (2011) Uncovering secrets behind low-resistance planing craft hull forms through optimization. Eng Optim 43(11):1161–1173. 10.1080/0305215X.2010.546841

[88] Bertetta D, Brizzolara S, Gaggero S, Viviani M, Savio L (2012) CPP propeller cavitation and noise optimization at different pitches with panel code and validation by cavitation tunnel measurements. Ocean Eng 53:177–195. 10.1016/j.oceaneng.2012.06.026

[89] Guha A, Falzaranoa J (2015) Application of multi objective genetic algorithm in ship hull optimization. Ocean Syst Eng 5(2):91–107. 10.12989/OSE.2015.5.2.091

[90] Gaggero S, Villa D, Tani G, Viviani M, Bertetta D (2017) Design of ducted propeller nozzles through a RANSE-based optimization approach. Ocean Eng 145:444–463. 10.1016/j.oceaneng.2017.09.037

[91] Bonfiglio L, Perdikaris P, Vernengo G, De Medeiros JS, Karniadakis G (2018) Improving SWATH seakeeping performance using multi-fidelity Gaussian process and Bayesian optimization. J Ship Res 62(04):223–240. 10.5957/JOSR.11170069

[92] Gaggero S, Vernengo G, Villa D, Bonfiglio L (2020) A reduced order approach for optimal design of efficient marine propellers. Ships Offshore Struct 15(2):200–214. 10.1080/17445302.2019.1606877

[93] Furcas F, Vernengo G, Villa D, Gaggero S (2020) Design of wake equalizing ducts using RANSE-based SBDO. Appl Ocean Res 97:102087. 10.1016/j.apor.2020.102087

[94] Maia HWS, Mounsif S, Hernández-Fontes JV, Silva R (2021) Computational fluid dynamics applied to river boat hull optimization. Marine Technol Soc J 55(5):94–108. 10.4031/MTSJ.55.5.9

[95] Kostas KV, Ginnis AI, Politis CG, Kaklis PD (2015) Ship-hull shape optimization with a T-spline based BEM-isogeometric solver. Comput Methods Appl Mech Eng 284:611–622. 10.1016/j.cma.2014.10.030

[96] Liu X, Zhang H, Liu Q, Dong S, Xiao C (2021) A cross-entropy algorithm based on Quasi-Monte Carlo estimation and its application in hull form optimization. Int J Naval Architect Ocean Eng 13:115–125. 10.1016/j.ijnaoe.2021.01.001

[97] Percival S, Hendrix D, Noblesse F (2001) Hydrodynamic optimization of ship hull forms. Appl Ocean Res 23(6):337–355. 10.1016/S0141-1187(02)00002-0

[98] Zakerdoost H, Ghassemi H, Ghiasi M (2013) An evolutionary optimization technique applied to resistance reduction of the ship hull form. J Naval Archit Marine Eng 10(1):1–12. 10.3329/jname.v10i1.12927

[99] Luo W, Lan L (2017) Design optimization of the lines of the Bulbous Bow of a hull based on parametric modeling and computational fluid dynamics calculation. Math Comput Appl 22(1):4. 10.3390/mca22010004

[100] Tomasz Abramowski, Karol Sugalski (2017) Energy saving procedures for fishing vessels by means of numerical optimization of hull resistance. Sci J Maritime Univ Szczecin 121(49):19–27

[101] Barbarić M, Guzović Z (2020) Investigation of the possibilities to improve hydrodynamic performances of micro-hydrokinetic turbines. Energies 13(17):4560. 10.3390/en13174560

[102] Ma Y, Bi H, Hu M, Zheng Y, Gan L (2019) Hard sail optimization and energy efficiency enhancement for sail-assisted vessel. Ocean Eng 173:687–699. 10.1016/j.oceaneng.2019.01.026

[103] Sarıöz E (2009) Inverse design of ship hull forms for seakeeping. Ocean Eng 36(17–18):1386–1395. 10.1016/j.oceaneng.2009.08.011

[104] Guo J, Zhang Y, Chen Z, Feng Y (2020) CFD-based multi-objective optimization of a waterjet-propelled trimaran. Ocean Eng 195:106755. 10.1016/j.oceaneng.2019.106755

[105] Pehlivan Solak H (2020) Multi-dimensional surrogate based aft form optimization of ships using high fidelity solvers. Brodogradnja 71(1):85–100. 10.21278/brod71106

[106] Duvigneau R, Visonneau M, Deng GB (2003) On the role played by turbulence closures in hull shape optimization at model and full scale. J Marine Sci Technol 8(1):11–25. 10.1007/s10773-003-0153-8

[107] Campana EF, Peri D, Tahara Y, Kandasamy M, Stern F (2009) Numerical optimization methods for ship hydrodynamic design. In: Day 1 Wed, October 21, pp. 011–001004. SNAME, Providence, Rhode Island, USA (2009). 10.5957/SMC-2009-013 . https://onepetro.org/SNAMESMC/proceedings/SMC09/1-SMC09/D011S001R004/465503

[108] Tahara Y, Peri D, Campana EF, Stern F (2011) Single- and multiobjective design optimization of a fast multihull ship: numerical and experimental results. J Marine Sci Technol 16(4):412–433. 10.1007/s00773-011-0137-y

[109] Li S, Zhao F, Ni Q-J (2013) Multiobjective optimization for ship hull form design using SBD technique. CMES 92(2):123–149

[110] Peri D, Diez M (2013) Ship optimization by globally convergent modification of PSO by a surrogate-based Newton method. Eng Comput 30(4):548–561. 10.1108/02644401311329361

[111] Li S-Z, Zhao F, Ni Q-J (2014) Bow and stern shape integrated optimization for a full ship by a simulation-based design technique. J Ship Res 58(2):83–96. 10.5957/JOSR.58.2.130008

[112] Diez M, Campana EF, Stern F (2015) Design-space dimensionality reduction in shape optimization by Karhunen-Loève expansion. Comput Methods Appl Mech Eng 283:1525–1544. 10.1016/j.cma.2014.10.042

[113] Garg N, Kenway GKW, Lyu Z, Martins JRRA, Young YL (2015) High-Fidelity Hydrodynamic Shape Optimization of a 3-D Hydrofoil. J Ship Res 59(4):209–226. 10.5957/JOSR.59.4.150046

[114] Wu J, Liu X, Zhao M, Wan D (2017) Neumann-Michell theory-based multi-objective optimization of hull form for a naval surface combatant. Appl Ocean Res 63:129–141. 10.1016/j.apor.2017.01.007

[115] Yang L, Li SZ, Zhao F, Ni QJ (2018) An integrated optimization design of a fishing ship hullform at different speeds. J Hydrodyn 30(6):1174–1181. 10.1007/s42241-018-0079-5

[116] He P, Filip G, Martins JRRA, Maki KJ (2019) Design optimization for self-propulsion of a bulk carrier hull using a discrete adjoint method. Comput Fluids 192:104259. 10.1016/j.compfluid.2019.104259

[117] Miao A, Wan D (2020) Hull form optimization based on an NM+CFD integrated method for KCS. Int J Comput Methods 17(10):2050008. 10.1142/S0219876220500085

[118] Ni Q, Ruan W, Li S, Zhao F (2020) Multiple speed integrated optimization design for a SWATH using SBD technique. J Mar Sci Technol 25(1):185–195. 10.1007/s00773-019-00640-5

[119] Wang P, Chen Z, Feng Y (2021) Many-objective optimization for a deep-sea aquaculture vessel based on an improved RBF neural network surrogate model. J Mar Sci Technol 26(2):582–605. 10.1007/s00773-020-00756-z

[120] Villa D, Furcas F, Pralits JO, Vernengo G, Gaggero S (2021) An effective mesh deformation approach for hull shape design by optimization. J Marine Sci Eng 9(10):1107. 10.3390/jmse9101107

[121] Demo N, Ortali G, Gustin G, Rozza G, Lavini G (2021) An efficient computational framework for naval shape design and optimization problems by means of data-driven reduced order modeling techniques. Bollettino dell’Unione Matematica Italiana 14(1):211–230. 10.1007/s40574-020-00263-4

[122] Khan S, Kaklis P (2021) From regional sensitivity to intra-sensitivity for parametric analysis of free-form shapes: application to ship design. Adv Eng Inform 49:101314. 10.1016/j.aei.2021.101314

[123] Demo N, Tezzele M, Mola A, Rozza G (2021) Hull shape design optimization with parameter space and model reductions, and self-learning mesh morphing. J Marine Sci Eng 9(2):185. 10.3390/jmse9020185

[124] Zhang S, Tezdogan T, Zhang B, Lin L (2021) Research on the hull form optimization using the surrogate models. Eng Appl Comput Fluid Mech 15(1):747–761. 10.1080/19942060.2021.1915875

[125] Yang C, Huang F, Kim H (2014) Hydrodynamic optimization of a triswach. J Hydrodyn 26(6):856–864. 10.1016/S1001-6058(14)60094-9

[126] Yang C, Huang F (2016) An overview of simulation-based hydrodynamic design of ship hull forms. J Hydrodyn 28(6):947–960. 10.1016/S1001-6058(16)60696-0

[127] Harries S, Uharek S (2021) Application of radial basis functions for partially-parametric modeling and principal component analysis for faster hydrodynamic optimization of a catamaran. J Marine Sci Eng 9(10):1069. 10.3390/jmse9101069

[128] Chang H, Zhan C, Liu Z, Cheng X, Feng B (2021) Dynamic sampling method for ship resistance performance optimisation based on approximated model. Ships Offshore Struct 16(4):386–396. 10.1080/17445302.2020.1730090

[129] Zheng Q, Feng B-W, Chang H-C, Liu Z-Y (2021) Dynamic space reduction optimization framework and its application in hull form optimization. Appl Ocean Res 114:102812. 10.1016/j.apor.2021.102812

[130] Nazemian A, Ghadimi P (2022) Shape optimisation of trimaran ship hull using CFD-based simulation and adjoint solver. Ships Offshore Struct 17(2):359–373. 10.1080/17445302.2020.1827807

[131] Zhang S-I, Zhang B-j, Tezdogan T, Xu L-p, Lai Y-y (2017) Research on bulbous bow optimization based on the improved PSO algorithm. China Ocean Eng 31(4):487–494. 10.1007/s13344-017-0055-9

[132] Tezdogan T, Shenglong Z, Demirel YK, Liu W, Leping X, Yuyang L, Kurt RE, Djatmiko EB, Incecik A (2018) An investigation into fishing boat optimisation using a hybrid algorithm. Ocean Eng 167:204–220. 10.1016/j.oceaneng.2018.08.059

[133] Zhang S, Tezdogan T, Zhang B, Xu L, Lai Y (2018) Hull form optimisation in waves based on CFD technique. Ships Offshore Struct 13(2):149–164. 10.1080/17445302.2017.1347231

[134] Nazemian A, Ghadimi P (2020) Automated CFD-based optimization of inverted bow shape of a trimaran ship: proposing an applicable and efficient optimization platform. Sci Iran 2020:56644

[135] Nazemian A, Ghadimi P (2021) CFD-based optimization of a displacement trimaran hull for improving its calm water and wavy condition resistance. Appl Ocean Res 113:102729. 10.1016/j.apor.2021.102729

[136] Tahara Y, Tohyama S, Katsui T (2006) CFD-based multi-objective optimization method for ship design. Int J Numer Meth Fluids 52(5):499–527. 10.1002/fld.1178

[137] Saha GK, Suzuki K, Kai H (2004) Hydrodynamic optimization of ship hull forms in shallow water. J Marine Sci Technol. 10.1007/s00773-003-0173-3

[138] Saha GK, Suzuki K, Kai H (2005) Hydrodynamic optimization of a catamaran hull with large bow and stern bulbs installed on the center plane of the catamaran. J Mar Sci Technol 10(1):32–40. 10.1007/s00773-004-0186-6

[139] Zhang B-J, Zhang S-L, Zhang H (2018) Optimization design of minimum total resistance hull form based on CFD method. China Ocean Eng 32(3):323–330. 10.1007/s13344-018-0033-x

[140] Hong ZC, Zong Z, Li HT, Hefazi H, Sahoo PK (2017) Self-blending method for hull form modification and optimization. Ocean Eng 146:59–69. 10.1016/j.oceaneng.2017.09.048

[141] Zong Z, Hong Z, Wang Y, Hefazi H (2018) Hull form optimization of trimaran using self-blending method. Appl Ocean Res 80:240–247. 10.1016/j.apor.2018.09.003

[142] Kandasamy M, Peri D, Ooi SK, Carrica P, Stern F, Campana EF, Osborne P, Cote J, Macdonald N, De Waal N (2011) Multi-fidelity optimization of a high-speed foil-assisted semi-planing catamaran for low wake. J Mar Sci Technol 16(2):143–156. 10.1007/s00773-011-0119-0

[143] Klanac A, Ehlers S, Jelovica J (2009) Optimization of crashworthy marine structures. Mar Struct 22(4):670–690. 10.1016/j.marstruc.2009.06.002

[144] Ehlers S (2010) A procedure to optimize ship side structures for crashworthiness. Proc Instit Mech Engi M: J Eng Maritime Environ 224(1):1–11. 10.1243/14750902JEME179

[145] D’Agostino D, Serani A, Diez M (2020) Design-space assessment and dimensionality reduction: an off-line method for shape reparameterization in simulation-based optimization. Ocean Eng 197:106852. 10.1016/j.oceaneng.2019.106852

[146] Li J-I, Wang X-j, Wang P, Dong H-c, Chen C-h (2021) Shape optimization for a conventional underwater glider to decrease average periodic resistance. China Ocean Eng 35(5):724–735. 10.1007/s13344-021-0064-6

[147] Hamed A (2022) Multi-objective optimization method of trimaran hull form for resistance reduction and propeller intake flow improvement. Ocean Eng 244:110352. 10.1016/j.oceaneng.2021.110352

[148] Bellman R (1957) Dynamic programming, 1st edn. Princeton University Press, Princeton

[149] Zhang H, Liu Z, Zhan C, Feng B (2016) A sensitivity analysis of a hull’s local characteristic parameters on ship resistance performance. J Mar Sci Technol 21(4):592–600. 10.1007/s00773-016-0378-x

[150] Geremia P, Maki KJ, Lavini G, Genuzio H (2012) Hull design method combining an innovative flow solver coupled with efficient multivariate analysis and optimization strategies. J Ship Product Design 28(4):164–171. 10.5957/JSPD.28.4.120057

[151] Chen X, Diez M, Kandasamy M, Zhang Z, Campana EF, Stern F (2015) High-fidelity global optimization of shape design by dimensionality reduction, metamodels and deterministic particle swarm. Eng Optim 47(4):473–494. 10.1080/0305215X.2014.895340

[152] Serani A, Leotardi C, Iemma U, Campana EF, Fasano G, Diez M (2016) Parameter selection in synchronous and asynchronous deterministic particle swarm optimization for ship hydrodynamics problems. Appl Soft Comput 49:313–334. 10.1016/j.asoc.2016.08.028

[153] Pellegrini R, Serani A, Liuzzi G, Rinaldi F, Lucidi S, Diez M (2020) Hybridization of multi-objective deterministic particle swarm with derivative-free local searches. Mathematics 8(4):546. 10.3390/math8040546

[154] Serani A, Fasano G, Liuzzi G, Lucidi S, Iemma U, Campana EF, Stern F, Diez M (2016) Ship hydrodynamic optimization by local hybridization of deterministic derivative-free global algorithms. Appl Ocean Res 59:115–128. 10.1016/j.apor.2016.04.006

[155] Liu X, Zhao W, Wan D (2021) Linear reduced order method for design-space dimensionality reduction and flow-field learning in hull form optimization. Ocean Eng 237:109680. 10.1016/j.oceaneng.2021.109680

[156] Doijode PS, Hickel S, Van Terwisga T, Visser K (2022) A machine learning approach for propeller design and optimization: Part I. Appl Ocean Res 124:103178. 10.1016/j.apor.2022.103178

[157] Serani A, Pellegrini R, Wackers J, Jeanson C-E, Queutey P, Visonneau M, Diez M (2019) Adaptive multi-fidelity sampling for CFD-based optimisation via radial basis function metamodels. Int J Comput Fluid Dyn 33(6–7):237–255. 10.1080/10618562.2019.1683164

[158] Pellegrini R, Serani A, Liuzzi G, Rinaldi F, Lucidi S, Diez M (2022) A derivative-free line-search algorithm for simulation-driven design optimization using multi-fidelity computations. Mathematics 10(3):481. 10.3390/math10030481

[159] Khan S, Kaklis P, Serani A, Diez M (2022) Geometric moment-dependent global sensitivity analysis without simulation data: application to ship hull form optimisation. Comput Aided Des 151:103339. 10.1016/j.cad.2022.103339

[160] Campana EF, Liuzzi G, Lucidi S, Peri D, Piccialli V, Pinto A (2009) New global optimization methods for ship design problems. Optim Eng 10(4):533–555. 10.1007/s11081-009-9085-3

[161] Brizzolara S, Curtin T, Bovio M, Vernengo G (2012) Concept design and hydrodynamic optimization of an innovative SWATH USV by CFD methods. Ocean Dyn 62(2):227–237. 10.1007/s10236-011-0471-y

[162] Danışman DB (2014) Reduction of demi-hull wave interference resistance in fast displacement catamarans utilizing an optimized centerbulb concept. Ocean Eng 91:227–234. 10.1016/j.oceaneng.2014.09.018

[163] Muratoglu A, Yuce MI (2017) Design of a river hydrokinetic turbine using optimization and CFD simulations. J Energy Eng 143(4):04017009. 10.1061/(ASCE)EY.1943-7897.0000438

[164] Li L, Jiang Z, Ong MC, Hu W (2019) Design optimization of mooring system: an application to a vessel-shaped offshore fish farm. Eng Struct 197:109363. 10.1016/j.engstruct.2019.109363

[165] Grigoropoulos GJ (2004) Hull form optimization for hydrodynamic performance. Marine Technol SNAME News 41(04):167–182. 10.5957/mt1.2004.41.4.167

[166] Subramanian R et al (2020) Genetic algorithm based design optimization of a passive anti-roll tank in a sea going vessel. Ocean Eng 203:107216. 10.1016/j.oceaneng.2020.107216

[167] Nazemian A, Ghadimi P (2021) Global optimization of trimaran hull form to get minimum resistance by slender body method. J Braz Soc Mech Sci Eng 43(2):67. 10.1007/s40430-020-02791-8

[168] Vesting F, Gustafsson R, Bensow RE (2016) Development and application of optimisation algorithms for propeller design. Ship Technol Res 63(1):50–69. 10.1080/09377255.2016.1145916

[169] Kostas KV, Ginnis AI, Politis CG, Kaklis PD (2017) Shape-optimization of 2D hydrofoils using an Isogeometric BEM solver. Comput Aided Des 82:79–87. 10.1016/j.cad.2016.07.002

[170] Chen J, Wei J, Jiang W (2016) Optimization of a twin-skeg container vessel by parametric design and CFD simulations. Int J Naval Archit Ocean Eng 8(5):466–474. 10.1016/j.ijnaoe.2016.05.008

[171] Cheng X, Feng B, Liu Z, Chang H (2018) Hull surface modification for ship resistance performance optimization based on Delaunay triangulation. Ocean Eng 153:333–344. 10.1016/j.oceaneng.2018.01.109

[172] Zheng Q, Chang H-C, Liu Z-Y, Feng B-W (2021) Application of dynamic space reduction method based on partial correlation analysis in hull optimization. J Ship Res 65(02):167–178. 10.5957/JOSR.04190019

[173] Han C, Kim H, Park S (2014) Optimal design of floating substructures for spar-type wind turbine systems. Wind Struct 18(3):253–265. 10.12989/WAS.2014.18.3.253

[174] Nazemian A, Ghadimi P (2022) A multi-objective optimisation study of trimaran hull applying RBF-Morph technique and integrated optimisation platform at two design speeds. Ships Offshore Struct 17(12):2628–2640. 10.1080/17445302.2021.2010442

[175] Poloni C, Giurgevich A, Onesti L, Pediroda V (2000) Hybridization of a multi-objective genetic algorithm, a neural network and a classical optimizer for a complex design problem in fluid dynamics. Comput Methods Appl Mech Eng 186(2–4):403–420. 10.1016/S0045-7825(99)00394-1

[176] Cirello A, Mancuso A (2008) A numerical approach to the keel design of a sailing yacht. Ocean Eng 35(14–15):1439–1447. 10.1016/j.oceaneng.2008.07.002

[177] Mahmood S, Huang D (2012) Computational fluid dynamics based bulbous bow optimization using a genetic algorithm. J Mar Sci Appl 11(3):286–294. 10.1007/s11804-012-1134-1

[178] Joung T-H, Sammut K, He F, Lee S-K (2012) Shape optimization of an autonomous underwater vehicle with a ducted propeller using computational fluid dynamics analysis. Int J Naval Archit Ocean Eng 4(1):44–56. 10.2478/IJNAOE-2013-0077

[179] Vasudev KL, Sharma R, Bhattacharyya SK (2014) A multi-objective optimization design framework integrated with CFD for the design of AUVs. Methods Oceanogr 10:138–165. 10.1016/j.mio.2014.08.002

[180] Chrismianto D, Kim D-J (2014) Parametric bulbous bow design using the cubic Bezier curve and curve-plane intersection method for the minimization of ship resistance in CFD. J Mar Sci Technol 19(4):479–492. 10.1007/s00773-014-0278-x

[181] Leifsson L, Hermannsson E, Koziel S (2015) Optimal shape design of multi-element trawl-doors using local surrogate models. J Comput Sci 10:55–62. 10.1016/j.jocs.2015.01.006

[182] Du W, Zhao Y, He Y, Liu Y (2016) Design, analysis and test of a model turbine blade for a wave basin test of floating wind turbines. Renew Energy 97:414–421. 10.1016/j.renene.2016.06.008

[183] Gao T, Wang Y, Pang Y, Cao J (2016) Hull shape optimization for autonomous underwater vehicles using CFD. Eng Appl Comput Fluid Mech 10(1):599–607. 10.1080/19942060.2016.1224735

[184] Alam K, Ray T, Anavatti SG (2017) Design optimization of an unmanned underwater vehicle using low- and high-fidelity models. IEEE Trans Syst Man Cybern Syst 47(11):2794–2808. 10.1109/TSMC.2015.2390592

[185] Mizzi K, Demirel YK, Banks C, Turan O, Kaklis P, Atlar M (2017) Design optimisation of propeller boss cap fins for enhanced propeller performance. Appl Ocean Res 62:210–222. 10.1016/j.apor.2016.12.006

[186] Halder P, Mohamed MH, Samad A (2018) Wave energy conversion: design and shape optimization. Ocean Eng 150:337–351. 10.1016/j.oceaneng.2017.12.072

[187] Zhang S, Zhang B, Tezdogan T, Xu L, Lai Y (2018) Computational fluid dynamics-based hull form optimization using approximation method. Eng Appl Comput Fluid Mech 12(1):74–88. 10.1080/19942060.2017.1343751

[188] Duvigneau R, Visonneau M (2004) Hydrodynamic design using a derivative-free method. Struct Multidisc Optim. 10.1007/s00158-004-0414-z

[189] Tahara Y, Peri D, Campana EF, Stern F (2008) Computational fluid dynamics-based multiobjective optimization of a surface combatant using a global optimization method. J Mar Sci Technol 13(2):95–116. 10.1007/s00773-007-0264-7

[190] Renaud P, Sacher M, Scolan Y-M (2022) Multi-objective hull form optimization of a SWATH configuration using surrogate models. Ocean Eng 256:111209. 10.1016/j.oceaneng.2022.111209

[191] Vernengo G, Bonfiglio L, Gaggero S, Brizzolara S (2016) Physics-based design by optimization of unconventional supercavitating hydrofoils. J Ship Res 60(4):187–202. 10.5957/JOSR.60.4.150074

[192] Berrini E, Mourrain B, Roux Y, Durand M, Fontaine G (2017) Geometric modelling and deformation for shape optimization of ship hulls and appendages. J Ship Res 61(02):91–106. 10.5957/jsr.2017.61.2.91

[193] Guerrero J, Cominetti A, Pralits J, Villa D (2018) Surrogate-based optimization using an open-source framework: the bulbous bow shape optimization case. Math Comput Appl 23(4):60. 10.3390/mca23040060

[194] Coppedè A, Gaggero S, Vernengo G, Villa D (2019) Hydrodynamic shape optimization by high fidelity CFD solver and Gaussian process based response surface method. Appl Ocean Res 90:101841. 10.1016/j.apor.2019.05.026

[195] Wang Y, Gao T, Pang Y, Tang Y (2019) Investigation and optimization of appendage influence on the hydrodynamic performance of AUVs. J Mar Sci Technol 24(1):297–305. 10.1007/s00773-018-0558-y

[196] Abdollahzadeh MJ, Moosavi A (2020) Optimization of microgrooves for water-solid drag reduction using genetic algorithm. J Ocean Engd Marine Energy 6(3):221–242. 10.1007/s40722-020-00170-y

[197] Wang Y, Joseph J, Aniruddhan Unni TP, Yamakawa S, Barati Farimani A, Shimada K (2022) Three-dimensional ship hull encoding and optimization via deep neural networks. J Mech Des 144(10):101701. 10.1115/1.4054494

[198] Ehlers S (2012) A particle swarm algorithm-based optimization for high-strength steel structures. J Ship Prod Design 28(01):1–9. 10.5957/jspd.2012.28.1.1

[199] Sun L, Wang D (2012) Optimal structural design of the midship of a VLCC based on the strategy integrating SVM and GA. J Mar Sci Appl 11(1):59–67. 10.1007/s11804-012-1106-5

[200] Dong H, Song B, Wang P (2017) Kriging-based optimization design for a new style shell with black box constraints. J Algorithms Comput Technol 11(3):234–245. 10.1177/1748301817709601

[201] Jia D, Li F, Zhang C, Li L (2019) Design and simulation analysis of trimaran bulkhead based on topological optimization. Ocean Eng 191:106304. 10.1016/j.oceaneng.2019.106304

[202] Dejhalla R, Mrša Z, Vuković S (2001) Application of genetic algorithm for ship hull form optimization. Int Shipbuild Prog 48(2):117–133

[203] Kitamura M, Uedera T (2003) Optimization of ship structure based on zooming finite element analysis with sensitivities. Int J Offshore Polar Eng 13(01):10

[204] Jang B-S, Ko D-E, Suh Y-S, Yang Y-S (2009) Adaptive approximation in multi-objective optimization for full stochastic fatigue design problem. Mar Struct 22(3):610–632. 10.1016/j.marstruc.2008.11.001

[205] Lee Y-T, Ahuja V, Hosangadi A, Ebert M (2010) Shape optimization of a multi-element foil using an evolutionary algorithm. J Fluids Eng 132(5):051401. 10.1115/1.4001343

[206] Brizzolara S, Vernengo G (2011) Automatic optimization computational method for unconventional SWATH ships resistance. Int J Math Models Methods Appl Sci 5(5):882–889

[207] Whitfield RI, Duffy AHB, Gatchell S, Marzi J, Wang W (2012) A collaborative platform for integrating and optimising computational fluid dynamics analysis requests. Comput Aided Des 44(3):224–240. 10.1016/j.cad.2011.04.004

[208] Lu Y, Chang X, Hu A-k (2016) A hydrodynamic optimization design methodology for a ship bulbous bow under multiple operating conditions. Eng Appl Comput Fluid Mech 10(1):330–345. 10.1080/19942060.2016.1159987

[209] Liu X, Zhao M, Wan D, Wu J (2017) Hull form multi-objective optimization for a container ship with Neumann-Michell theory and approximation model. Int J Offshore Polar Eng 27(4):423–432. 10.17736/ijope.2017.mmr18

[210] Rotteveel E, Hekkenberg R, Van Der Ploeg A (2017) Inland ship stern optimization in shallow water. Ocean Eng 141:555–569. 10.1016/j.oceaneng.2017.06.028

[211] Wang SM, Ma S, Duan WY (2018) Seakeeping optimization of trimaran outrigger layout based on NSGA-II. Appl Ocean Res 78:110–122. 10.1016/j.apor.2018.06.010

[212] Fu X, Lei L, Yang G, Li B (2018) Multi-objective shape optimization of autonomous underwater glider based on fast elitist non-dominated sorting genetic algorithm. Ocean Eng 157:339–349. 10.1016/j.oceaneng.2018.03.055

[213] Vasudev KL, Sharma R, Bhattacharyya SK (2018) Shape optimisation of an AUV with ducted propeller using GA integrated with CFD. Ships Offshore Struct 13(2):194–207. 10.1080/17445302.2017.1351292

[214] Tahara Y, Ichinose Y, Kaneko A, Kasahara Y (2019) Variable decomposition approach applied to multi-objective optimization for minimum powering of commercial ships. J Mar Sci Technol 24(1):260–283. 10.1007/s00773-018-0551-5

[215] Cheng X, Feng B, Chang H, Liu Z, Zhan C (2019) Multi-objective optimisation of ship resistance performance based on CFD. J Mar Sci Technol 24(1):152–165. 10.1007/s00773-018-0543-5

[216] Vasudev KL, Sharma R, Bhattacharyya SK (2019) Multi-objective shape optimization of submarine hull using genetic algorithm integrated with computational fluid dynamics. Proc Instit Mech Eng M: J Eng Maritime Environ 233(1):55–66. 10.1177/1475090217714649

[217] Luo Y, Pan G, Huang Q, Shi Y, Lai H (2019) Parametric geometric model and shape optimization of airfoils of a biomimetic manta ray underwater vehicle. J Shanghai Jiaotong Univ (Sci) 24(3):402–408. 10.1007/s12204-019-2076-4

[218] Kõrgesaar M, Ehlers S (2010) An assessment procedure of the crashworthiness of an LNG tanker side structure. Ship Technol Res 57(2):108–119. 10.1179/str.2010.57.2.003

[219] De Pina AA, Albrecht CH, De Lima BSLP, Jacob BP (2011) Tailoring the particle swarm optimization algorithm for the design of offshore oil production risers. Optim Eng 12(1–2):215–235. 10.1007/s11081-009-9103-5

[220] Yin X, Lu Q, Lu Y, Zou J, Wan L (2021) Hydrodynamic optimization of foreship hull-form using contrastive optimization algorithms. J Coast Res 37(5):1063–1078

[221] Li S, Zhu F, Hou X, Ni Q (2022) Application of mesh deformation and adaptive method in hullform design optimization. J Mar Sci Technol 27(1):566–575. 10.1007/s00773-021-00851-9

[222] Alam K, Ray T, Anavatti SG (2014) Design and construction of an autonomous underwater vehicle. Neurocomputing 142:16–29. 10.1016/j.neucom.2013.12.055

[223] Huang F, Yang C (2016) Hull form optimization of a cargo ship for reduced drag. J Hydrodyn 28(2):173–183. 10.1016/S1001-6058(16)60619-4

[224] Campana EF, Diez M, Iemma U, Liuzzi G, Lucidi S, Rinaldi F, Serani A (2016) Derivative-free global ship design optimization using global/local hybridization of the DIRECT algorithm. Optim Eng 17(1):127–156. 10.1007/s11081-015-9303-0

[225] Cinquini C, Venini P, Nascimbene R, Tiano A (2001) Design of a river-sea ship by optimization. Struct Multidisc Optim 22(3):240–247. 10.1007/s001580100141

[226] Tahara Y, Stern F, Himeno Y (2004) Computational fluid dynamics-based optimization of a surface combatant. J Ship Res 48(04):273–287. 10.5957/jsr.2004.48.4.273

[227] Choi HJ (2015) Hull-form optimization of a container ship based on bell-shaped modification function. Int J Naval Archit Ocean Eng 7(3):478–489. 10.1515/ijnaoe-2015-0034

[228] Baoji Z (2020) Research on ship hull optimisation of high-speed ship based on viscous flow/potential flow theory. Polish Maritime Res 27(1):18–28. 10.2478/pomr-2020-0002

[229] Kunasekaran M, Rhee SH, Venkatesan N, Samad A (2021) Design optimization of a marine current turbine having winglet on blade. Ocean Eng 239:109877. 10.1016/j.oceaneng.2021.109877

[230] Bonfiglio L, Perdikaris P, Brizzolara S (2020) Multi-fidelity Bayesian optimization of SWATH hull forms. J Ship Res 64(02):154–170. 10.5957/jsr.2020.64.2.154

[231] Sarıöz E (2012) Minimum ship size for seakeeping. Proc Instit Mech Eng M: J Eng Maritime Environ 226(3):214–221. 10.1177/1475090212440068

[232] Tran TG, Van Huynh C, Kim HC (2021) Optimal design method of bulbous bow for fishing vessels. Int J Naval Archit Ocean Eng 13:858–876. 10.1016/j.ijnaoe.2021.10.006

[233] Tran TG, Van Huynh Q, Kim HC (2022) Optimization strategy for planing hull design. Int J Naval Archit Ocean Eng 14:100471. 10.1016/j.ijnaoe.2022.100471

[234] Kröger J, Kühl N, Rung T (2018) Adjoint volume-of-fluid approaches for the hydrodynamic optimisation of ships. Ship Technol Res 65(1):47–68. 10.1080/09377255.2017.1411001

[235] Yu L, Druckenbrod M, Greve M, Wang K-Q, Abdel-Maksoud M (2015) Numerical and experimental analysis of a ducted propeller designed by a fully automated optimization process under open water condition. China Ocean Eng 29(5):733–744. 10.1007/s13344-015-0051-x

[236] Turan O, Cui H (2012) A reinforcement learning based hybrid evolutionary algorithm for ship stability design. In: Chiong R, Weise T, Michalewicz Z (eds) Variants of evolutionary algorithms for real-world applications. Springer, Berlin, pp 281–303. 10.1007/978-3-642-23424-8_9

[237] Ge Z, Korpus R, Shen Z (2016) Optimization of stern-tube bearing performance by CFD-based fluid-structures interaction. SNAME maritime convention. SNAME, pp 033–015003

[238] Nazemian A, Ghadimi P (2023) Simulation-based multi-objective optimization of side-hull arrangement applied to an inverted-bow trimaran ship at cruise and sprint speeds. Eng Optim 55(2):214–235. 10.1080/0305215X.2021.1993843

[239] Lee M, Cho S-G, Choi J-S, Kim H-W, Hong S, Lee TH (2012) Metamodel-based multidisciplinary design optimization of a deep-sea manganese nodules test miner. J Appl Math 2012:1–18. 10.1155/2012/326954

[240] Huang R, Dai Y, Luo X, Wang Y, Huang C (2019) Multi-objective optimization of the flush-type intake duct for a waterjet propulsion system. Ocean Eng 187:106172. 10.1016/j.oceaneng.2019.106172

[241] Thandayutham K, Avital EJ, Venkatesan N, Samad A (2019) Optimization of a horizontal axis marine current turbine via surrogate models. Ocean Syst Eng 9(2):111–133. 10.12989/OSE.2019.9.2.111

[242] Miao A, Zhao M, Wan D (2020) CFD-based multi-objective optimisation of S60 Catamaran considering Demihull shape and separation. Appl Ocean Res 97:102071. 10.1016/j.apor.2020.102071

[243] Liu X, Zhao W, Wan D (2021) Hull form optimization based on calm-water wave drag with or without generating bulbous bow. Appl Ocean Res 116:102861. 10.1016/j.apor.2021.102861

[244] Cairns J, Vezza M, Green R, MacVicar D (2021) Numerical optimisation of a ship wind-assisted propulsion system using blowing and suction over a range of wind conditions. Ocean Eng 240:109903. 10.1016/j.oceaneng.2021.109903

[245] Liu X-w, Zhao W-w, Wan D-c (2021) Optimization of the roll motion of box-shaped hull section with anti-rolling sloshing tanks and fins in beam waves. J Hydrodyn 33(4):688–697. 10.1007/s42241-021-0067-z

[246] Liu Z, Zhao W, Wan D (2022) Resistance and wake distortion optimization of JBC considering ship-propeller interaction. Ocean Eng 244:110376. 10.1016/j.oceaneng.2021.110376

[247] Qiu W, Song X, Shi K, Zhang X, Yuan Z, You Y (2019) Multi-objective optimization of semi-submersible platforms using particle swam optimization algorithm based on surrogate model. Ocean Eng 178:388–409. 10.1016/j.oceaneng.2019.02.039

[248] Wu S-J, Lin C-C, Liu T-L, Su I-H (2020) Robust design on the arrangement of a sail and control planes for improvement of underwater Vehicle’s maneuverability. Int J Naval Archit Ocean Eng 12:617–635. 10.1016/j.ijnaoe.2020.06.002

[249] Lin Y, Yang Q, Guan G (2019) Automatic design optimization of SWATH applying CFD and RSM model. Ocean Eng 172:146–154. 10.1016/j.oceaneng.2018.11.044

[250] Yang Q, Lin Y, Guan G (2020) Improved sequential sampling for meta-modeling promotes design optimization of SWATH. Ocean Eng 198:106958. 10.1016/j.oceaneng.2020.106958

[251] Sun T, Chen G, Yang S, Wang Y, Wang Y, Tan H, Zhang L (2021) Design and optimization of a bio-inspired hull shape for AUV by surrogate model technology. Eng Appl Comput Fluid Mech 15(1):1057–1074. 10.1080/19942060.2021.1940287

[252] Guan G, Yang Q, Wang Y, Zhou S, Zhuang Z (2021) Parametric design and optimization of SWATH for reduced resistance based on evolutionary algorithm. J Mar Sci Technol 26(1):54–70. 10.1007/s00773-020-00721-w

[253] Feng Y, Chen Z, Dai Y, Cui L, Zhang Z, Wang P (2022) Multi-objective optimization of a bow thruster based on URANS numerical simulations. Ocean Eng 247:110784. 10.1016/j.oceaneng.2022.110784

[254] Lv H, Wei C, Liang X, Yi H (2022) Optimisation of wave-piercing trimaran outrigger layout with comprehensive consideration of resistance and seakeeping. Ocean Eng 250:111050. 10.1016/j.oceaneng.2022.111050

[255] Guan G, Wang L, Geng J, Zhuang Z, Yang Q (2021) Parametric automatic optimal design of USV hull form with respect to wave resistance and seakeeping. Ocean Eng 235:109462. 10.1016/j.oceaneng.2021.109462

[256] Yang M, Wang Y, Chen Y, Wang C, Liang Y, Yang S (2022) Data-driven optimization design of a novel pressure hull for AUV. Ocean Eng 257:111562. 10.1016/j.oceaneng.2022.111562

[257] Xu L, Li P, Qin H (2021) Optimization of hydrodynamic performance of ocean bottom flying node. Int J Offshore Polar Eng 31(04):403–410

[258] Thandayutham K, Samad A (2020) Hydrostructural optimization of a marine current turbine through multi-fidelity numerical models. Arab J Sci Eng 45(2):935–952. 10.1007/s13369-019-04185-y

[259] Gaggero S, Vernengo G, Villa D (2022) A marine propeller design method based on two-fidelity data levels. Appl Ocean Res 123:103156. 10.1016/j.apor.2022.103156

[260] Liu X, Zhao W, Wan D (2022) Multi-fidelity Co-Kriging surrogate model for ship hull form optimization. Ocean Eng 243:110239. 10.1016/j.oceaneng.2021.110239

[261] Di Fiore, F., Nardelli, M. & Mainini, L. Active Learning and Bayesian Optimization: A Unified Perspective to Learn with a Goal. Arch Computat Methods Eng (2024). 10.1007/s11831-024-10064-z

[262] Spinosa E, Pellegrini R, Posa A, Broglia R, De Biase M, Serani A (2023) Simulation-driven design optimization of a destroyer-type vessel via multi-fidelity supervised active learning. J Marine Sci Eng 11(12):2232. 10.3390/jmse11122232

[263] Valorani M, Peri D, Campana EF (2003) Sensitivity analysis methods to design optimal ship hulls. Optim Eng 4(4):337–364. 10.1023/B:OPTE.0000005391.23022.3b

[264] Zhang B-j, Ma K, Ji Z-s (2009) The optimization of the hull form with the minimum wave making resistance based on rankine source method. J Hydrodyn 21(2):277–284

[265] Wilson W, Hendrix D, Gorski J (2010) Hull form optimization for early stage ship design. Naval Eng J 122(2):53–65

[266] Zhang B-j (2012) Shape optimization of bow bulbs with minimum wave-making resistance based on Rankine source method. J Shanghai Jiaotong Univ (Sci) 17(1):65–69. 10.1007/s12204-012-1239-3

[267] Lv X, Wu X, Sun J, Tu H (2013) Trim optimization of ship by a potential-based panel method. Adv Mech Eng 5:378140. 10.1155/2013/378140

[268] Dambrine J, Pierre M, Rousseaux G (2016) A theoretical and numerical determination of optimal ship forms based on Michell’s wave resistance. ESAIM Control Optim Calculus Var 22(1):88–111. 10.1051/cocv/2014067

[269] Ignacio LC, Victor RR, Francisco DRR, Pascoal A (2019) Optimized design of an autonomous underwater vehicle, for exploration in the Caribbean Sea. Ocean Eng 187:106184. 10.1016/j.oceaneng.2019.106184

[270] Page BR, Mahmoudian N (2020) Simulation-driven optimization of underwater docking station design. IEEE J Oceanic Eng 45(2):404–413. 10.1109/JOE.2018.2885200

[271] Chen Y, Liu Y, Liu W, Ge Y, Xue Y, Zhang L (2022) Optimal design of radial inflow turbine for ocean thermal energy conversion based on the installation angle of nozzle blade. Renew Energy 184:857–870. 10.1016/j.renene.2021.12.016

[272] Wang K, Luo G, Li Y, Xia R, Liu H (2020) Multi-condition optimization and experimental verification of impeller for a marine centrifugal pump. Int J Naval Archit Ocean Eng 12:71–84. 10.1016/j.ijnaoe.2019.07.002

[273] Zhu D, Tao R, Lu Z, Wu Y, Xiao R (2022) Optimization design of the internal structural support of marine turbine blade for weight reduction: A preliminary study. Ocean Eng 260:111989. 10.1016/j.oceaneng.2022.111989

[274] Lemmer F, Yu W, Müller K, Cheng PW (2020) Semi-submersible wind turbine hull shape design for a favorable system response behavior. Mar Struct 71:102725. 10.1016/j.marstruc.2020.102725

[275] Jang B-S, Kim JD, Park T-Y, Jeon SB (2019) FEA based optimization of semi-submersible floater considering buckling and yield strength. Int J Naval Archit Ocean Eng 11(1):82–96. 10.1016/j.ijnaoe.2018.02.010

[276] Feng Y, El Moctar O, Schellin TE (2021) Parametric hull form optimization of containerships for minimum resistance in calm water and in waves. J Mar Sci Appl 20(4):670–693. 10.1007/s11804-021-00243-w

[277] Zhang B-J, Zhang C, She W-X (2020) The minimum wave resistance of hull form design method based on CFD method. J Ship Prod Design 36(03):161–170. 10.5957/JSPD.09180036

[278] Zha L, Zhu R, Hong L, Huang S (2021) Hull form optimization for reduced calm-water resistance and improved vertical motion performance in irregular head waves. Ocean Eng 233:109208. 10.1016/j.oceaneng.2021.109208

[279] Goren O, Calisal SM, Bulent Danisman D (2017) Mathematical programming basis for ship resistance reduction through the optimization of design waterline. J Mar Sci Technol 22(4):772–783. 10.1007/s00773-017-0447-9

[280] Zhao C, Wang W (2021) Optimisation of hull form of ocean-going trawler. Brodogradnja 72(4):33–46

[281] Timurlek H, Şener B (2022) Hydrodynamic optimization of a high-speed vessel by means of simulation-based design methodology. Proc Instit Mech Eng M: J Eng Maritime Environ 236(4):891–903. 10.1177/14750902221091345

[282] Fitriadhy A, Rizat NS, Abd Razak AR, Abdullah SF, Mahmuddin F, Kurniawan A (2022) Optimization modelling of a catamaran hull form towards reducing ship total resistance. CFD Lett 14(4):67–79

[283] Wang SM, Duan WY, Xu QL, Duan F, Deng GZ, Li Y (2021) Study on fast interference wave resistance optimization method for trimaran outrigger layout. Ocean Eng 232:109104. 10.1016/j.oceaneng.2021.109104

[284] Nazemian A, Ghadimi P (2022) Multi-objective optimization of ship hull modification based on resistance and wake field improvement: combination of adjoint solver and CAD-CFD-based approach. J Braz Soc Mech Sci Eng 44(1):27. 10.1007/s40430-021-03335-4

[285] Hou Y-H, Jiang X-J, Shi X-H (2017) Ship hull optimization based on new neural network. J Comput 28(1):137–148. 10.3966/199115592017022801011

[286] Jiang J-W, Qi J-T, Cai H-P, Chen K, Huang W-X (2020) Prediction and optimisation of low-frequency discrete- and broadband-spectrum marine propeller forces. Appl Ocean Res 98:102114. 10.1016/j.apor.2020.102114

[287] Ha Y, Kim W, Cho S (2006) Design sensitivity analysis and topology optimization method applied to stiffener layout in hull structures. J Ship Res 50(03):222–230. 10.5957/jsr.2006.50.3.222

[288] Serani A, Diez M (2023) Parametric model embedding. Comput Methods Appl Mech Eng 404:115776. 10.1016/j.cma.2022.115776

